# Co-designing zoonotic diseases prevention practices when people depend on wild meat

**DOI:** 10.1016/j.onehlt.2025.101074

**Published:** 2025-05-13

**Authors:** Natacha Efoua Tomo, Aude Pouliquen, Gilles Aurélien Boupana Mapeyi, Patrice Makouloutou-Nzassi, Nadine N’dilimabaka, Barthélémy Ngoubangoye, Daniel Cornelis, Sébastien Lebel, Marisa Peyre, Marie-Marie Olive, Hadrien Vanthomme, Gael Darren Maganga, Alexis Delabouglise

**Affiliations:** a Unité Émergence des Maladies Virales, Département de Virologie, Centre Interdisciplinaire de Recherches Médicales de Franceville (CIRMF), Franceville, Gabon; b CIRAD, UMR ASTRE, 34398 Montpellier, France; c ASTRE, Université de Montpellier, CIRAD, INRAE, Montpellier, France; d Institut de Recherche Agronomique et Forestière (IRAF), Libreville, Gabon; e Institut de Recherches en Ecologie Tropicale (IRET), Libreville, Gabon; f Département de Biologie, Université des Sciences et Techniques de Masuku (USTM), BP 901 Franceville, Gabon; g Centre de Primatologie (CDP), Centre Interdisciplinaire de Recherches Médicales de Franceville (CIRMF), Franceville, Gabon; h CIRAD, UPR Forêts et Sociétés, 34398 Montpellier, France; i Forêts et Sociétés, Université de Montpellier, CIRAD, Montpellier, France; j Institut d'Agronomie et de Biotechnologies (INSAB), Université des Sciences et Techniques de Masuku (USTM), BP 901 Franceville, Gabon

**Keywords:** One health, Zoonoses, Biosecurity, Wildlife, Wild meat, Hunting, Central Africa

## Abstract

In the face of the escalating frequency of diseases emergences originating from wildlife, the development of reliable strategies for controlling zoonotic diseases transmission at the interface between wildlife and human is becoming a global priority. Rural communities whose subsistence is based on hunting for wild meat extraction are natural targets of such interventions, because of their regular contacts with wildlife. To date there have been few attempts at building preventive sanitary strategies taking into account the socioeconomic and institutional constraints in which wild meat systems operate. The study presented here, conducted in eastern Gabon, aimed at conceiving risk-reduction strategies of zoonotic diseases transmitted from wildlife in a two-phase approach, namely (1) an assessment phase, based on a survey on risk knowledge and practices conducted with members of communities living on wild meat, and (2) a co-design phase based on focus group discussions to identify acceptable prevention strategies aimed at limiting the contacts creating the major risks of exposure to zoonoses infections. The use of participatory methods aiming at eliciting issues and solutions from the participants, enabled the conception of strategies that were adapted to the context and well accepted by stakeholders at different stages, namely the track, capture, transport of wild animals, the butchering of carcasses, cooking and consumption process. However, some limitations to the effective application of the strategies can be anticipated notably because of (1) the current low and biased perception of zoonotic risks by wild meat actors, and (2) the economic incentives for maintaining risky behaviors like the capture and trade of live animals and the consumption or sale of animals found dead or displaying signs of disease infection.

## 1 Introduction

Zoonotic diseases are thought to have accounted for 60 % of infectious disease emergencies since the middle of the twentieth century, most often with wildlife as the primary reservoir or intermediary host [[1], [2], [3]]. These diseases pose significant threats to human health worldwide, as demonstrated by the Ebola outbreaks in Africa [[4], [5], [6]] and the recent SARS-CoV-2 pandemic [7,8]. The devastating impacts of these diseases highlight the critical need for robust preventive strategies to mitigate their emergence and spread [9]. Contacts between humans and wildlife driven by activities related to hunting and wild meat trade are considered a major risk for pathogen spillover between animals and humans by international organizations [[10], [11]]. In the rural communities surrounding forested regions in Central and West Africa, hunting and wild meat trade represent a critical mean of subsistence, providing both an income and high-protein foods essential for their nutrition [[12], [13], [14], [15]]. However, these activities also place these communities at heightened risk of exposure to zoonotic disease transmission due to close contacts with wild animals and their products, at different stages of the supply chain [[16], [17], [18], [19]]. The past Ebola outbreaks highlighted this risk, since the initial transmission from wildlife to humans was frequently attributed to the capture of infected non-human primates [[20], [21], [22], [23]].

While stringent regulatory measures on wildlife hunting and trade can have detrimental effects on the livelihood of the rural communities and be ultimately counterproductive [24], the promotion of safe practices of wild animals hunting and handling is considered a promising lever for reducing the risk of diseases emergence attributable to wild meat [25]. However limited research has been conducted on safe practices that communities can readily implement at minimal cost, and current recommended biosafety measures often fail to account for the socio-economic constraints faced by individuals [11]. These constraints may include limited financial resources, restricted access to information, and deeply rooted cultural practices. In addition, several studies have indicated that individuals involved in the wild meat trade have a limited knowledge of zoonotic risks and an uneven perception of the importance of these risks for their own safety [[26], [27], [28], [29]]. To be effective, prevention strategies must be adapted to the realities of these communities and incorporate practical and accessible solutions that respect their livelihood context and specific needs. In this regard, the application of participatory approaches allows for the collection of valuable information on the context in which local actors operate, with elicitation methods specifically designed for this purpose [30,31]. Additionally, the active involvement of local actors in the design and prioritisation of health protection measures was successfully performed with other topics, and had a positive effect on their acceptability and engagement of community members [[32], [33], [34]].

The present study aimed at identifying priority risk-reduction practices that can be implemented by communities whose livelihood depends on wild meat trade and consumption to limit the risk of zoonotic disease transmission at the human-wildlife interface. It proceeded in two distinct phases, namely (1) an assessment of the knowledge and practices of community members focusing on exposure to zoonotic risks at the interface with wildlife, using a standardized questionnaire, and (2) a co-design of acceptable risk-reduction strategies with the hunting community members, using a participatory approach.

## 2 Method

### 2.1 Study area and target population

The study targeted the communities practicing wildlife hunting in the department of Mulundu, in the province of Ogooué-Lolo in Gabon, central Africa. Hunting is practised year-round in Mulundu for the trade and home consumption of wild meat. It is a primary source of protein and income for the rural population, and it is not prohibited but regulated [35]. The most commonly hunted animals are duikers, porcupine, red river hogs and monkeys. The survival of these commonly hunted species does not seem to be threatened due to the relatively low level of hunting pressure compared to the ecosystem productivity.

### 2.2 Selection of participating communities

Eleven communities known for practicing hunting were selected for the study. Each of these communities were composed of several villages. The 11 communities were chosen according to a set of criteria in order to incorporate the diversity of geographic and socioeconomic contexts of the department. (1) The communities were distributed over the three main road axes of the Mulundu department, six communities benefiting from an asphalt road and access to the mobile phone network, and five others being only connected by forest roads. A proper connection to transport and communication facilities possibly favors the access to both wild meat market outlets and health services and information, with potential implications for wild meat activities and health protection behaviors. (2) Three communities had medical clinics accessible to the population, while eight communities only had access to a distant health centre only accessible by car, which possibly affected their respective use of health services and level of awareness on health risks. (3) Finally, eight of the selected communities participated in the Sustainable Wildlife Management (SWM)^2^ program, a EU-funded program promoting communities' sustainable hunting, while three communities did not. The project intervention consisted in setting up hunter associations, legal frameworks for hunting activities and sustainable hunting plans, and therefore affected the hunting and wild meat trading practices of the involved communities. Following the assessment phase, a subset of five communities out of 11 were selected from the initial sample for inclusion in the co-design phase. These communities all participated in the SWM program but were representative of the diversity of contexts, according to the aforementioned criteria (1) and (2): location on the three main road axes, diversity of access to communication and transport networks and medical facilities.

### 2.3 Assessment of the current knowledge and practices

A questionnaire-based survey on zoonotic risk knowledge and practices was administered to members of the 11 communities in order to assess their hunting and handling practices of wild animals' and their products as well as their perception and knowledge of zoonotic risks associated with these activities. The questionnaire was divided into nine sections namely (1) general information on the respondent; practices associated with the (2) hunting; (3) transport; (4) butchering; (5) storing; (6) cooking of wild animals; (7) contacts between wild and domestic animals; (8) actions taken when discovering suspect deaths (i.e. death with no apparent reason) or signs of sickness in wild and domestic animals; (9) perception and knowledge of sanitary risks associated with the contact with animals. Respondents systematically completed sections 1, 7, 8, and 9, while sections 2 to 6 were completed only by participants who had been involved in the corresponding activity within the 12 months before the interview. The questionnaire is available in Appendix A. Questionnaires were all administered by the first author of the article using the KoboCollect application (https://www.kobotoolbox.org/). Every member of the selected communities who were known to engage in activities related to hunting were offered to be interviewed. These people were identified with the help of the presidents of community hunting associations and the local authorities. During the interview, the hunters were asked to list the people who took part in transporting, butchering, storing and cooking the products of their hunt within the last 12 months. These people were, in turn, offered to be interviewed.

### 2.4 Co-design of risk reduction practices

**Fig. 1:**
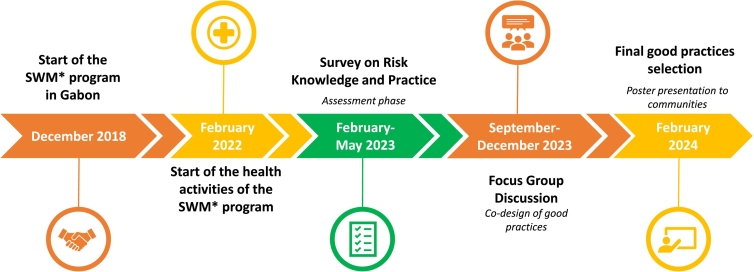
Timeline of implementation of the research activities in the study area of Mulundu.

A series of focus group discussions (FGDs) was conducted with members of the five selected communities in order to identify practices that community members could implement in the future to reduce their exposure to zoonotic disease transmission during hunting or manipulation of animals or wildlife products. A preliminary list of risky practices to be discussed with participants was established by the investigators. The practices were selected among the ones reported by the respondents to the survey on risk knowledge and practices. The investigators prioritized in their selection the risky contacts most frequently reported by the questionnaire respondents (e.g. injuries during butchering, transport of live animals), as well as some less frequently reported contacts considered to generate a particularly high risk of exposure to pathogens known to circulate in the region (e.g. bite by live animals and rabies). One male FGD (with only male participants) and one female FGD (with only female participants) were conducted by two FGD facilitators in each of the five pilot communities. It was assumed that this separation would allow women to freely express their opinion without being influenced or intimidated by male community members. Community members were informed of the FGDs beforehand by the presidents of the hunting associations and volunteers presented themselves at the interview sites. The objectives of the interview were presented to the participating community members before starting the discussion. The pre-identified risky practices were presented one by one. For each of the listed risky practices, participants proposed risk reduction solutions. The solutions were written on individual and anonymous yellow post-it notes subsequently displayed on a board visible to everyone. They were read out one by one by the facilitators to all participants. Each solution was then discussed in order to identify its advantages, disadvantages and the feasibility of its implementation. Participants wrote these advantages and disadvantages individually and anonymously on post-it notes with specific color codes (pink: disadvantages; green: advantages). These post-it notes were then placed next to the corresponding solutions on the common board. The resulting solution tree, including risky practices, proposed alternatives and their associated advantages and disadvantages, was presented to the participants for discussion and validation. The FGDs were entirely conducted in French language. Participants with a limited literacy level were assisted by the facilitators to write their contribution on the post-it notes and those who could not properly understand or speak French were assisted by volunteer schoolchildren who acted as translators. The timeline of activities is illustrated in Fig. 1.

### 2.5 Data material and analysis

The data generated by the questionnaire survey on risk knowledge and practices were stored in the investigators' professional laptops. The dataset was cleaned and analysed with simple descriptive statistics and graphs generated using R version 3 [36]. The data generated by the co-design phase consisted of the notes taken and audio records from the FGDs. They were transcribed into electronic format text and the audio records were destroyed within three months after the interviews. We performed a content analysis for identifying pre-defined elements of interest contained in the transcripts, namely (1) discussed risky practices, (2) alternative solutions, or and (3) advantages and disadvantages cited for each alternative solution. The analysis proceeded in the following steps: (1) Transcripts were read and elements of interest were identified and listed in an Excel spreadsheet; (2) Solutions displaying significant similarities were merged; (3) advantages and disadvantages attributed to each solution were listed.

## 3 Results

### 3.1 Assessment of the current risk knowledge and practices

The questionnaire was tested with eight respondents and the final version was administered to 309 respondents, including 192 (62 %) males and 117 (38 %) females. 177 (57 %) participants were involved in hunting, 212 (69 %) in transporting, 240 (78 %) in wild animal butchering, 287 (93 %) in animal product storing, and 289 (94 %) in animal products cooking. Male and female participation was reported in all activities except hunting, which was reserved to male. 16 persons declined the interview proposition, mostly due to a lack of time.

**Fig. 2:**
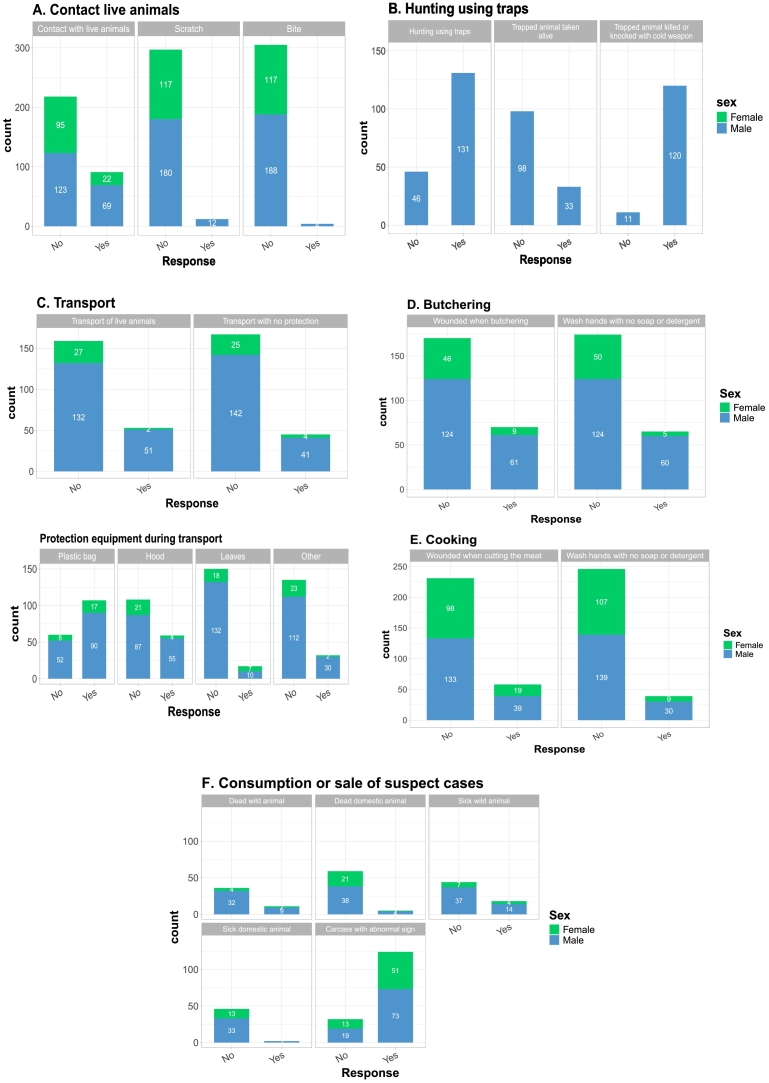
Bar plot sex-differentiated representation of the frequency of responses of participants to the questionnaire survey on practices at risk of zoonotic disease transmission in the past 12 months. White color numbers on the bars correspond to numbers of respondents while black color percentage numbers above the bars indicate the proportion of questioned respondents having reported the considered risky practice. Listed practices include (A) the contacts with live animals and the occurrence of bites or scratches, (B) hunting of animals with traps and the condition of capture of trapped animals, (C) conditions of transport of animals and the equipment used for it that protect the transporter from direct contact with animals and its fluids, (D) the butchering of wild animal carcasses, (E) the cooking of wild animal meat, (F) the consumption or sell of wild or domestic animals displaying a sign of diseases suspicion (death of unknown reason, clinical signs or abnormal lesion sign on the carcass).

The frequency of reporting of risky practices among respondents is reported in a sex-disaggregated form in Fig. 2. Direct contacts with live wild animals were frequently reported by respondents (29 %, *n* = 91), more often by males (36 %) compared to females (19 %). However, relatively few instances of bites (*n* = 4) or scratches (*n* = 12), were reported. Hunting with traps, which increases the risk of contacts by hunters with live animals, was reported by 75 % (*n* = 131) of the hunters. 26 % (*n* = 33) of the hunters using traps reported capturing a fraction of the trapped animals alive and 91 % (*n* = 120) reported killing or stunning them with a weapon (stick, machete, or spear), exposing them to direct contacts with the animals or their bloods and to potential aggressions. 25 % (*n* = 53) of respondents involved in animal transportation reported transporting live animals, creating further risk of exposure to live animal contacts. 96 % of these respondents (*n* = 51) were males. For 90 % of them (*n* = 48) this practice was motivated by the high demand of their customers for live animals. 21 % of respondents (*n* = 45) involved in transportation reported using no particular equipment to protect themselves from direct contacts with the transported animals. Among those who reported the use of an equipment, 64 % used plastic bags, 35 % used hoods, and 10 % used leaves, these materials being often used in combination. 29 % (*n* = 70) of the respondents practising butchering reported having been wounded during the process at least once over the past year (33 % of the males and 16 % of the females) and 27 % (*n* = 65) reported using no soap or detergent when washing their hands after butchering (33 % of the males and 9 % of the females). 20 % (*n* = 58) of respondents involved in the cooking of wild animal products reported having been wounded during the process at least once over the past year (23 % of the males and 16 % of the females) and 14 % (*n* = 39) reported using no soap or detergent when washing their hands (18 % of the males and 8 % of the females). Among respondents who experienced the discovery of dead wild animals, dead domestic animals, live wild animals displaying clinical signs of illness, live domestic animals displaying clinical signs, and abnormal lesions in wild or domestic animal carcasses 23 %, 8 %, 29 %, 4 %, and 79 % reported consumption or sale of the concerned animals respectively.

**Fig. 3:**
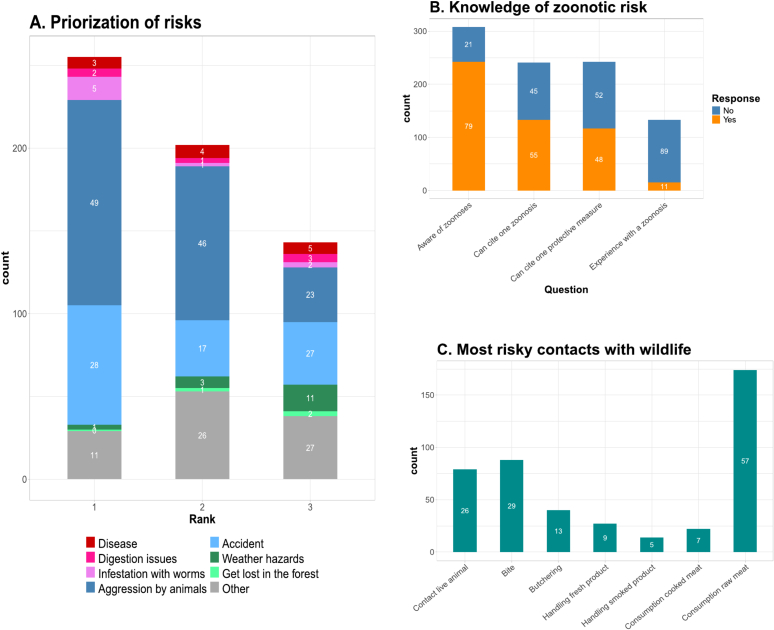
Bar plot representation of the frequencies of responses of participants to the questionnaire survey on perception and knowledge of zoonotic risks. White color numbers on the bars correspond to corresponding percentages. (A) Perceived risks associated with hunting and handling wild animals ranked as first, second and third in terms of importance; (B) answers to specific questions aimed at assessing the awareness, knowledge and experience of participants on zoonotic risks; (C) Contacts between wildlife and humans considered as most risky for health by participants.

Respondents were asked to name up to three risks associated with activities related to hunting and wild meat and to rank them by order of importance. Their responses are reported in Fig. 3A: 304, 293, and 288 respondents were able to name a first ranking, second ranking and third ranking risk respectively. Aggression by a wild animal and accidents linked to the manipulation of wild animals or the hunting equipment were reported by 54 % and 40 % of the respondents respectively, either as a first, second or third ranking risk. In comparison sanitary issues, including disease infection, digestive problem or infestation of the meat with worms were reported as one of the risks by 6 %, 5 % and 6 % of respondents respectively. When asked whether they believed diseases could be transmitted from animals to humans, 79 % (*n* = 242) of respondents gave a positive answer (Fig. 3B). Among them, 55 % (*n* = 133) were able to cite at least one disease and 48 % (*n* = 117) were able to cite at least one measure to protect themselves from zoonotic disease infection. The zoonotic diseases most frequently cited by respondents included Ebola (*n* = 76), coronaviruses (*n* = 14), rabies (n = 14), and avian influenza (*n* = 10). Among the ones who were able to cite at least one disease, 11 % (*n* = 15) reported knowing someone or having heard about someone who had a past experience of disease infection attributed to contacts with animals (Fig. 3B). Most participants (57 %) cited consumption of raw meat as a major at-risk contact with wildlife that could affect the health of community members, while other contacts were more rarely put forward as major risks, like bite (29 %), direct contact with wild animals (26 %), or butchering of animals (13 %) (Fig. 3C). Additional results concerning the storage of wild meat products, disposal of unconsumed parts and perception of zoonotic diseases prevention are available in Appendix B.

### 3.2 Co-design of risk reduction practices

107 participants from 10 FGDs contributed to the identification of alternatives to risky practices. The risky contacts pre-selected by the investigators included (1) exposure to bite or scratch during hunting, (2) capture of live animals during hunting, (3) transport of live animals, (4) direct contact with body and fluids of animals during transport, (5) contact with body or fluids of the animals during butchering, (6) rick of injuries during butchering, (7) consumption of animals displaying clinical signs of diseases or abnormal carcass lesions, (8) consumption of animals found dead for no apparent reason.

The risk-reducing alternatives identified by participants during FGDs are listed in Table 1. Most of the suggested alternatives involved no or minimal financial cost to the community members and made use of resources available in the forest (e.g. wood or leaves) or recycled material that communities use in their daily lives like plastic bags and wraps. To limit the risk associated with bites and scratches by wild animals during hunting, the most frequently cited solution consisted in killing the animal properly before manipulation (number of citations *n* = 40). Participants considered the hunter could kill the animal in different ways: with a rifle, machete, spear or wooden stick when it is trapped, rifle being the safest way of killing. Some participants suggested waiting a sufficient time before approaching (*n* = 37) and using two times the usual amount of rifle cartridge to ensure that the animal is definitely killed. However, this strategy increases the number of used cartridges and the associated cost. Participants suggested adapting the amount of cartridge to the animal size since the carcass of small animals, such as a porcupine, can be destroyed by an excessive number of shots, making them unsuitable for sale and consumption. The machete was considered more efficient at ensuring a definitive kill than a wooden stick but it increased the risk of exposure of the user to bites and scratches because of its small size.

“It's easy to shoot an animal, to kill an animal in a trap, especially a small animal, with an adapted weapon” (community B, male group).

“If it's a wild boar, I can finish it off [with two cartridges] and I'll make a lot of money [from the sale of game]” (community D, male group).

**Table 1:** List of propositions of alternative practices to reduce the risk of disease transmission from animals to humans at different stages, from animal hunting to consumption.

		Cumulative number of citations	Number of FGDs where the risks was discussed	Number of FGDs where the solution was cited
Risk of Bite and Scratch	HUNTING			
	Killing the animal properly	40	5	3
	Ensure that the animal is dead before approaching it	37	5	5
	Personal Protective Equipment	15	5	4
	No hunting	1	5	1
	CAPTURE AND TRANSPORT OF LIVE ANIMALS			
	Suitable capture and manipulation technique	43	4	4
	Transport with solid plastic packaging	12	4	4
	Do not capture live animals	10	4	2
	Avoid direct contact with the animal	2	4	2
	Take precautions (no more precisions)	2	4	1
	Carry on the shoulder with a wood stick	1	4	1
	Walk fast to reduce transport duration	1	4	1

	TRANSPORT OF DEAD ANIMALS			
Contact with Animal Body Fluids	Use a plastic bag	42	8	8
	Use leaves	28	8	7
	Use plastic wrap	27	8	7
	Use a solid plastic bag (flour or rice bag, backpack, military bag)	13	8	7
	Use a hood	7	8	6
	Use a basket	4	8	4
	Personal Protective Equipment	4	8	3
	Carry on the shoulder with a wood stick	3	8	3
	Do not transport	2	8	1
	Combine leaves, bag and hood	2	8	2
	Combine plastic wrap and leaves	1	8	1
	Use a bucket	1	8	1
	Use a wheelbarrow	1	8	1
	Smoke animals before transporting	1	8	1

	BUTCHERING AND PREPARATION			
Injuries During Butchering and Cooking	Take precautions	89	9	9
	Proper positioning of the carcass	6	9	4
	Leave a space between the hand and the knife blade	5	9	1
	Use a special knife	3	9	2
	Butcher in the river	1	9	1
	Wear personal protective equipment	1	9	1
Contact with Animal Body Fluids During Butchering and Cooking	Use soap to wash hands	40	9	8
	Use lukewarm or hot water to wash hands	22	9	7
	Wear gloves	8	9	5
	Use bleach to wash hands	6	9	4
	Use pharmaceutical alcohol or alcoholic beverage to wash hands	4	9	3
	Avoid open wounds	3	9	1
	Obtain help from another person (to avoid injury)	3	9	3
	Use sand in addition to water to wash hands	1	9	1
	Use diesel fuel to wash hands	1	9	1
	Wash meat and inspect its appearance	1	9	1

	SUSPECT CASES			
Handling of Animal with Clinical Signs, Abnormalities or Dead for No Apparent Reason	Leave the animal	35	9	9
	Do not touch	25	9	7
	Contact a representative	15	9	4
	Do not eat or cook	12	9	7
	Smoke the meat	5	9	1
	Cover with leaves to avoid consumption by other animals	5	9	2
	If transported to the village, throw away	4	9	4
	Eat	4	9	2
	Show to the husband	2	9	2
	When a woman buys or is offered a wild animal product, she asks under what conditions it was hunted to avoid consuming a suspect animal	2	9	1
	Keep out of the way	2	9	2
	Incinerate	1	9	1
	Check for signs of traps or guns	1	9	1
	Bury	1	9	1
	Feed the dog with the carcass	1	9	1

Wearing clothing that protects the hunter's skin was also deemed effective at limiting the risk of bites and scratches (*n* = 15). All hunters reported having hunting clothes, but their quality depended on the financial means at their disposal. Some participants pointed out their inadequacy with certain hunting techniques that require being as uncovered as possible in order to limit the noise made when approaching the animal.

“It's easy for workers and those who can afford it to buy boots, gloves and so on” (community C, male group).

“There is a lack of protective equipment on the local market” (community D, male group).

“it's noisy! and it's heavy, especially when it's wet, it makes noise. Boum boum boum!” (community C, male group).

Only some of the participating hunters captured live animals. Participants noted that capturing and restraining live animals requires a suitable level of dexterity (*n* = 43). Various techniques are used: the hunter can break off limbs that can cause injuries (e.g. legs, horns), block the legs or tie the animal up with ropes. For transporting live animals, participants considered it was safer to use solid equipment such as a hood, backpack or plastic bag (*n* = 12). According to some participants the only effective solution is not to capture and transport live animals at all (*n* = 10). Someone suggested transporting live animals by attaching them to a wood stick that the carrier can take upon his shoulders (n = 1) and participants agreed it is possible for a short distance and a small animal. Other people proposed to walk fast to reduce transport duration (n = 1).

“If you're not a professional, you can't come into contact with an animal brought to the village alive” (community E, male group).

“It's difficult because when the animal is alive, it jumps, it's very nasty and you can get hurt” (community D, male group).

Placing the animal carcass in a plastic bag (*n* = 42) or packing it with leaves (*n* = 28), a plastic wrap (*n* = 27), a solid plastic bag (rice bag, backpack or military bag; *n* = 13) or a plastic bag with leaves inside (n = 2) were deemed good solutions to protect the carcass carrier from direct contact with body fluids. Some participants pointed out that packing the carcass with plastic material can accelerate the putrefaction process, making the carcass unfit for consumption. Leaves are easy to find in the forest and help to preserve the freshness of the transported carcass but are not hermetic and can tear up during transport. Leaves can also be used as vegetable gutters to drain fluids from the carrying bag, a practice reportedly used by elderly women. Participants agreed that the best way to avoid contact with body fluids while preserving the freshness of the meat was to use plastic bags combined with leaves. Regardless of the packing process, it was considered the packed carcass needed to be placed in a basket (*n* = 4) or a hood (*n* = 7) for ease of transport. Some participants suggested wearing personal protective equipment (n = 4) or several layers of clothes to prevent contact with body fluids of dead animals but it was considered ineffective at protecting the carrier in case of abundant body fluid.

To avoid injuries during butchering it was most often recommended to take precautions: working slowly in a quiet place, sharpening the knife well, and staying focused (*n* = 89). While many participants considered injuries are easily avoided with some practice, some of the female participants recognized experiencing distractions that can lead to injuries, such as interruption by children or phone calls. In order to reduce contacts with body fluids, participants most commonly suggested using soap and water (*n* = 40) to wash their hands. Soap is available in all households and at low cost. However, hunters reported that it attracts midges and, during hunting, the smell of soap scares away animals. Simple water washing was also suggested (*n* = 22). According to hunters it is easy to find water in the forest rivers. However, if they are located far away from a river and carry a heavy game, it may be difficult for them to wash their hands with water. The use of gloves was suggested too (*n* = 8). Other minor suggestions included the use of bleach (*n* = 6) or alcohol (n = 4), but these are rare and expensive commodities. Several people suggested avoiding butchering when having an open wound (*n* = 3) and, instead, asking someone else for help (n = 3).

“It's difficult to be careful and take good care of the game and not hurt yourself, sometimes someone or the kids can talk with you, they can call you suddenly, sometimes even the phone rings” (community E, female group).

“I wash my hands in hot water after butchering to remove the dirt so that the microbe doesn't touch me” (community E, female group).

“Soap often isn't prudent in hunting because even the smell of soap can scare off the animal you want to hunt” (community D, male group).

The proposed alternatives to the consumption or sale of the animals found sick or dead for no apparent reasons were to leave the animal (*n* = 35) or avoid contact (*n* = 25). However, some admitted that it is not easy to leave an animal found dead for no apparent reason, as it could feed the family or generate an income. Some participants pointed out that they themselves may buy or consume wild animals found in similar occasions, and stressed the need for all actors in the value chain to be made aware of the issue. Informing a community leader of the discovery (*n* = 15), not eating or cooking the animal (*n* = 12), or smoking the meat to kill the microbes before consumption (*n* = 5) were also suggested. One participant suggested inspecting the game for signs of traps or cartridges (n = 1) to determine whether the death had been caused by a hunter. This suggestion was rejected by others, on the ground that dead animal can be contaminated with pathogens transmissible to humans.

“We experienced an Ebola disease when we were in Makokou. We were told that the disease had come because people had picked up a dead animal. They ate it, and that's why they were sick” (community B, female group).

“If nobody informs me that the game has been found dead for no apparent reason, I'll prepare it” (community C, female group).

A poster summarising the main recommendations arising from the study, selected by the investigators, was printed and permanently put on display in all pilot communities (Appendix C). The selection was made by the facilitators and based on two main criteria: (1) feasibility of implementation by hunting communities, according to the FGD results, and (2) effectiveness of the promoted measures at reducing the risk of transmission of zoonotic pathogens, according to the investigators' own expertise.

## 4 Discussion

More than ever, the global community is faced with the imperative of developing sustainable preventive strategies of zoonotic spillover at the interface between wildlife, human and domestic animals that are compatible with the environmental and socioeconomic contexts. Wild meat actors of West and Central Africa are highly exposed to zoonotic infection risks as their activity brings them into close contact with wildlife potentially infected with transmissible zoonotic diseases. They also have limited access to veterinary and medical facilities, their living areas being characterized by a low density of humans and domestic animals.

The limited perception of zoonotic risks among wild meat actors constitutes a major challenge to interventions aimed at promoting safe practices. A large majority of interviewees were aware of the possibility of disease transmission from animals to humans. Ebola was the most cited zoonotic disease, probably because of past outbreaks having affected the neighbouring Ogooué-Ivindo province [21,37]. Nevertheless, few respondents included zoonotic diseases in the main risks related to their activity and those risks were very marginally considered in comparison with aggression by wild animals or accidents. A low consideration for zoonotic risks among wild meat value chain actors was reported in other field studies in Sub-Saharan Africa [26,38]. It significantly differs from the observations commonly made in livestock systems [39,40], where animal diseases rank high as sources of concerns for farmers, not so much because of their potential public health implications but rather because of their resulting production losses associated with animal morbidity and mortality. While the hunted wildlife of the region is a known or potential host of zoonotic pathogens like Ebola virus [21,37], Marburg virus [41,42], Crimean Congo haemorrhagic fever virus [43] and Rift Valley fever virus [44], the actual level of exposure of wild meat actors to zoonotic diseases has not been thoroughly evaluated. The perception of this exposure level by community members is subject to bias, since zoonotic diseases in humans are most likely under-diagnosed and people are unlikely to establish a link between the occurrence of sickness in themselves or their relatives and previous contacts with animals. As a matter of fact, only a small fraction of the respondents could cite a previous experience of disease caused by contacts with animals among the people they knew. Noteworthily, participants most often considered the consumption of raw wild meat as a particularly risky contact for their health and attributed a relatively lower importance to direct contacts with live animals or to contacts with the carcass during the butchering process. This contradicts the scientific evidences that contacts with live animals constitute major pathways of exposure to zoonotic disease infection, in particular bites and scratches [45,46]. Additionally, the butchering of animals or the cooking of their products exposes individuals to infection risks through direct contacts with tissues and fluids [19]. This risk is elevated if the person in charge of butchering or meat preparation is wounded during the process and can be substantially reduced if hand washing is systematically performed afterwards with soap or detergent [47]. Actors with a low perception of sanitary risks may, logically, not attribute a high benefit to the adoption of safe practices, including avoidance of contacts with live animals, protection from contacts with animal carcasses and fluids and proper hand washing after carcass manipulation. A fraction of the respondents to the questionnaire survey reported the consumption or sale of animals found dead or displaying clinical symptoms or carcasses with lesions. For those actors, abandoning suspect animals may represent a loss of revenue or food for their family, while the anticipated benefit associated with zoonotic prevention might be perceived as low [30,48].

Wild meat practices exposing actors to zoonotic risks were mostly found in male hunting community members, who are involved in all activities related to wild meat, from hunting to consumption. Nevertheless, our results suggest that the role of women needs to be taken into account, since they occasionally transport animals during hunting trips and frequently butcher or cook wild animal products. They are therefore likely to observe signs of disease suspicions, notably carcass lesions. They also are seemingly more frequently implementing preventive measures, like proper hand washing with soap or detergent, than their male counterparts. However, female participants reported constraints specific to their familial duties that are also reported in other studies [[49], [50], [51]] and that may hinder the proper application of protective measures - for example the constant attention that childcare requires from them.

While implementing adapted communication campaigns to enhance zoonotic risk awareness among wild meat actors appears to be a necessary step forward, it may not be sufficient to establish a sustainable engagement of all actors, in the face of economic impediments. The co-design phase of our study partly addressed this obstacle by identifying low-cost, practical and acceptable alternatives to risky practices while fostering the engagement of community members in the risk-reduction process. Most of the proposed risk-reducing alternatives identified by participants are in line with biosecurity measures promoted by Wegner et al. [25] while being adapted to the living conditions and resources available to the communities. In particular, the alternatives most often put forward by groups of participants do not involve the purchase of new or expensive equipment but rather make use of material that households can easily obtain from their environment. The compatibility of some of the proposed alternatives, like the use of protective clothes, with the conditions and requirements of hunting activities, was also discussed. The implemented participatory process was instrumental to the efficient identification of the risk-reduction alternatives. In addition, the collective elaboration of the solutions through interactions between participants developed a sense of ownership that will positively influence future behaviour changes. The method could be replicated with communities in other geographical settings with distinct ecological or socioeconomic features in order both to expend the engagement of wild meat actors in biosecurity, and to assess the potential degree of diversity of co-designed solutions and the extent of their dependency to the local context.

The successful application of the co-designed risk reduction strategies will be dependent on the individual engagement of each community member, and some of them may be difficult to put in place for economic reasons. One example is the capture and transport of animals alive, motivated by demand from consumers willing to pay a higher price for live animals compared to carcasses. Similarly, not all hunters would readily forego the revenues from the sale of animals found sick or dead [48]. A previous study conducted specifically on hunters' willingness to report animals displaying signs of sickness to a fictive health surveillance authority revealed that this willingness was lower among hunters highly dependent on wild meat for their livelihood compared to hunters with more diversified activities [52].

Our study has some limitations. First, responses to the survey on risk knowledge and practices may have been affected by social desirability bias, leading to an underestimation of the true prevalence of risky practices among wild meat actors. Second, the communities taking part in the co-design of strategies were all participating in the SWM program. This selection bias may have influenced the results, since those communities were probably already more inclined to implement changes in their habits compared to other ones. It is therefore questionable to what extent the identified risk reduction strategies would be reproducible elsewhere.

## Authors contributions

NET, AP and AD conceived the study protocol (conceptualization), processed and analysed the data, and wrote the original manuscript. NET, AP, AD and MMO designed the questionnaires (methodology). NET and AP collected the data. AD supervised the study. AD, GDM and HV administered the project. DC, SL and MP secured the necessary funding for the study. GABM, PM-N, NN, BN, HV and GDM reviewed the manuscript.

## Funding

This study was carried out as part of the Sustainable Wildlife Management (SWM) Programme (https://www.swm-programme.info/), an initiative of the Organization of the African, Caribbean, and Pacific States funded by the 10.13039/501100000780European Union, with co-funding from the French Facility for Global Environment (FFEM) and the 10.13039/501100011061French Development Agency (AFD). The SWM Programme is being implemented by a consortium partnership, which includes the Food and Agriculture Organization of the United Nations (FAO), the French Agricultural Research Centre for International Development (CIRAD), the 10.13039/501100001836Centre for International Forestry Research (CIFOR) and the 10.13039/100005997Wildlife Conservation Society (WCS).

## Ethical approval and consent to participate

The study was implemented in accordance with the General Data Protection Regulation (GDPR) (2016/679) of the European Union concerning the collection and use of personal data. The data collected during the individual interviews and FGDs were stored in secured databases only accessible to the study investigators. Before their inclusion in the study, the investigators explained orally to the participants the objectives of the research, the type of information collected, the measures set to safeguard their privacy and their legal rights. Their consent to participate in the study and for allowing investigators to take notes and audio recordings was elicited orally. Because of the limited literacy rate, a written informed consent could not be obtained from each individual participant. Instead, an informed consent form containing the information listed above and a list of the names of the people who agreed to participate was read and signed by a representative of the community (the head of the community or the president of the community-based hunting association). The consent forms used in the different phases of the study are available in Appendix D. The study protocol was reviewed and approved by the Comité National d'Ethique en matière de Recherche Scientifique (CNERS) of Gabon (n°0021/2024/MESRSIT/CNERS/PR/SG).

## CRediT authorship contribution statement

**Natacha Efoua Tomo:** Writing – review & editing, Writing – original draft, Methodology, Investigation, Formal analysis, Data curation. **Aude Pouliquen:** Writing – review & editing, Writing – original draft, Methodology, Investigation, Formal analysis, Data curation. **Gilles Aurélien Boupana Mapeyi:** Writing – review & editing, Resources, Project administration, Investigation. **Patrice Makouloutou-Nzassi:** Supervision. **Nadine N’dilimabaka:** Supervision. **Barthélémy Ngoubangoye:** Writing – review & editing, Supervision. **Daniel Cornelis:** Project administration, Funding acquisition. **Sébastien Lebel:** Funding acquisition. **Marisa Peyre:** Project administration, Funding acquisition. **Marie-Marie Olive:** Methodology, Conceptualization. **Hadrien Vanthomme:** Writing – review & editing, Project administration. **Gael Darren Maganga:** Writing – review & editing, Validation, Supervision, Project administration. **Alexis Delabouglise:** Writing – original draft, Supervision, Project administration, Methodology, Formal analysis, Conceptualization.

## Declaration of competing interest

The authors declare that they have no known competing financial interests or personal relationships that could have appeared to influence the work reported in this paper.

## Data Availability

Data will be made available on request.

## Appendix Supplementary data

**Appendix A:** Questionnaire - Assessment of the knowledge and practices on zoonotic risks among wild meat actors. Image 1

**Appendix B:** Additional results of the questionnaire survey on zoonotic risk knowledge and practices. Image 2

**Appendix C:** Poster displaying the main recommendations to reduce the risk of disease transmission from animals to humans. Image 3

**Appendix D:** Informed consent forms. Image 4

## Footnotes

^2^ https://www.swm-programme.info/fr/homepage
