# Supplementary material for: Co-designing zoonotic diseases prevention practices when people depend on wild meat
Source: One Health. 2025 May 13;20:101074. doi: 10.1016/j.onehlt.2025.101074 (PMC12152596; doi:10.1016/j.onehlt.2025.101074)
Supplement: Supplementary file 1 — Appendix A: Questionnaire - Assessment of the knowledge and practices on zoonotic risks among wild meat actors. [file mmc1.pdf]

## Appendix A: Questionnaire - Assessment of the practices, knowledge and perception of zoonotic risks of wild meat system actors

### Table 1. List of questions

| Type | Name | Label | Condition of inclusion |
|---|---|---|---|
| start | start | | |
| end | end | | |
| begin_group | Interview_context | Survey background | |
| select_one cs5fx91 | Carrier_name | Name of interviewer(.rice) | |
| text | specify | specify | ${Packer_Name} = 'other |
| select_one qw1pg64 | Province | Province | |
| text | specify_001 | specify | ${Province} = 'other |
| select_one sz6zl15 | Department | Department | |
| text | precisez_002 | specify | ${Department} = 'other |
| select_one tu6wg63 | Community | Community | |
| text | precisez_003 | specify | ${Grouping} = 'other |
| select_one vb0bw83 | Village_BEMBICANI | Village (BEMBICANI) | ${Grouping} = 'bembicani |
| text | precisez_005 | specify | ${Village_BEMBICANI} = 'other |
| select_one oc52s06 | Village_MALENDE | Village (MALENDE) | ${Grouping} = 'malende |
| text | precisez_006 | specify | ${Village_MALENDE} = 'other |
| select_one ef2hh35 | Village_BOUNDZOUMBA | Village (BOUNDZOUMBA) | ${Grouping} = 'boundzoumba' |
| text | precisez_007 | specify | ${Village_BOUNDZOUMBA} = 'other |
| select_one pm6xo35 | Village_LIPAKA2 | Village (LIPAKA2) | Grouping} = 'lipaka_2 |
| text | precisez_008 | specify | ${Village_LIPAKA2} = 'other |
| select_one bp9sl17 | Village_KESSIPOUGHOU | Village (KESSIPOUGHOU) | ${Grouping} = 'kessipoughou' |
| text | precisez_009 | specify | ${KESSIPOUGHOU_Village} = 'other |
| select_one al8mf60 | Village_BAKOUSSOU_NDEKABALAND | Village (BAKOUSSOU-NDEKABALANDJI) | ${Grouping} = 'bakoussou_ndekabalandji' |
| text | specify_010 | specify | ${Village_BAKOUSSOU_NDEKABALAND} = 'other |
| select_one eg3qv46 | Village_BAPOSSO | Village (BAPOSSO) | ${Grouping} = 'baposso |
| text | precisez_011 | specify | ${Village_BAPOSSO} = 'other |

| | | | |
|---|---|---|---|
| select_one xh62m65 | Village_BOUNDZI | Village (BOUNDZI) | ${Grouping} = 'boundzi' |
| text | precisez_012 | specify | ${Village_BOUNDZI} = 'other |
| select_one cf2dr77 | Village_NZELA | Village (NZELA) | ${Grouping} = 'nzela' |
| text | precisez_013 | specify | ${NZELA_Village} = 'other |
| select_one pc1sf31 | Village_KOKOMOUNGUELE | Village (KOKOMOUNGUELE) | ${Grouping} = 'kokomounguele' |
| text | precisez_014 | specify | ${KOKOMOUNGUELE_Village} = 'other |
| select_one lc7tt73 | Village_MANA_MANA | Village (MANA-MANA) | ${Grouping} = 'mana_mana |
| text | precisez_014 | specify | ${Village_MANA_MANA} = 'other |
| date | Date | Date : | |
| end_group | | | |
| begin_group | Information_repondant | Respondent information | |
| text | Name | Name | |
| text | First name | First name | |
| select_one zp92d01 | Gender | Gender | |
| integer | Age | Age | |
| select_one py01n08 | Nationality | Nationality | |
| text | precisez_015 | specify | ${Nationality} = 'other |
| select_one qq07e03 | Ethnic group | Ethnic group | |
| text | precisez_016 | specify | ${Ethnia} = 'other |
| select_one pc8fd16 | Location_residence | Place of residence | |
| text | precisez_017 | specify | ${Lieu_residence} = 'other |
| select_one ta21o25 | Civil_Status | Civil status | |
| text | precisez_018 | specify | ${Civil_Status} = 'other |
| select_one cj4yc09 | Children | Do you have children? | |
| integer | Number_of_children | give the number | ${Children} = 'yes |
| select_one zh5ym19 | Study_level | What is your level of education | |
| text | precisez_019 | specify | ${Study_level} = 'other |
| select_multiple mo75z76 | Activities | Over the past 12 months, have you taken part in the following activities (please complete the following sections according to the answers given to this question)? | |
| end_group | | | |
| begin_group | Hunting | Hunting (as practiced over | selected(${Activities}, |

| | | | |
|---|---|---|---|
| | | the past 12 months) | 'hunting') |
| select_one dz9jk21 | Hunting_principal_activity | Over the last 12 months, is hunting your main activity? | |
| select_one nf7mv96 | Main_activity | What's your main business? | ${Main_hunt_activity} = 'no'. |
| text | precisez_020 | specify | ${Main_activity} = 'other'. |
| select_multiple hg7rt38 | Reason_hunting | What are your reasons for hunting (multiple answers possible)? | |
| text | precisez_021 | specify | selected(${Reason_Hunt}, 'other') |
| select_multiple gb6ts02 | Product_Sales | Who do you sell your hunting products to? | selected(${Reason_Hunt}, 'sell') |
| text | precisez_022 | specify | selected(${Product_Sales}, 'other') |
| select_one gh8ou58 | Hunting_income_importance | How important is hunting to your household income? | |
| select_multiple ww4pi43 | Species_hunting | What species do you hunt? | |
| text | precisez_023 | specify | selected(${Species_Hunt}, 'other') |
| select_one ia2gk86 | Chasse_davantage | Is there a period when you hunt more? | |
| select_multiple pr3sf30 | More_reason_hunting | Why? | ${More_hunt} = 'yes'. |
| text | precisez_024 | specify | selected(${Chase_more_reason}, 'other') |
| select_multiple qo1lb58 | Chasse_technique | What hunting technique do you use (multiple answers possible)? | |
| text | precisez_025 | specify | selected(${Technical_Hunt}, 'other') |
| select_multiple au3ky88 | Animal_vivant_piege | How do you catch the live animal in the trap/net? | selected(${Hunt_technique}, 'trap') or selected(${Hunt_technique}, 'net') |
| text | precisez_026 | specify | selected(${Animal_vivant_piege}, 'other') |
| select_multiple vy0ic01 | Chasse_piege_lieu | Where are you trap hunting? | selected(${Technical_Hunt}, 'trap') |
| text | precisez_027 | specify | selected(${Bait_hunt_place}, 'other') |
| select_multiple vy0ic01 | Rifle_hunting_place | Where do you hunt with a rifle? | selected(${Chasse_technique}, 'rifle') |
| text | precisez_028 | specify | selected(${Rifle_hunt_place}, 'other') |
| select_multiple np6ge53 | Chasse_piege_temps | When do you hunt for traps? | selected(${Technical_Hunt}, 'trap') |
| select_multiple np6ge53 | Time_gun_hunter | When do you go shotgun hunting? | selected(${Chasse_technique}, 'rifle') |
| select_one | Chasse_piege_sorties | How many trap hunting trips | selected(${Technical_Hunt}, |

| | | | |
|---|---|---|---|
| ay35j48 | | do you make per week? | 'trap') |
| select_one bq7gm71 | Chasse_piege_duree | What is the average length of your trap hunting trips? | selected(${Technical_Hunt}, 'trap') |
| select_one ay35j48 | Rifle_hunting_outlets | How many hunting trips do you take with your rifle each week? | selected(${Chasse_technique}, 'rifle') |
| select_one bq7gm71 | Rifle_hunting_duratione | What is the average length of your gun-hunting outings? | selected(${Chasse_technique}, 'rifle') |
| select_one gf7rz42 | Chasse_campement | Do you make hunting camp? | selected(${Chasse_technique}, 'rifle') |
| select_one an8zq70 | Chasse_prise_sorties | How many are taken on each hunting trip? | |
| select_multiple sp3nh12 | Repartition_prise_1 | How are these plugs distributed? (1) | ${Outlet_Chase} = '1' |
| select_multiple sp3nh12 | Repartition_prise_2 | How are these plugs distributed? (2) | ${Outlet_Chase} = '2' |
| select_multiple sp3nh12 | Repartition_prise_3 | How are these plugs distributed? (3) | ${Outlet_Chase} = '3' |
| select_multiple sp3nh12 | Repartition_prise_4 | How are these plugs distributed? (4) | ${Outlet_Chase} = '4' |
| select_multiple sp3nh12 | Repartition_prise_5 | How are these plugs distributed? (5) | ${Outlet_Chase} = '5' |
| select_multiple sp3nh12 | Repartition_prise_6 | How are these plugs distributed? (6) | ${Outlet_Chase} = '6' |
| select_multiple sp3nh12 | Repartition_prise_7 | How are these plugs distributed? (7) | ${Chasse_prise_sorties} = '7' |
| select_multiple sp3nh12 | Repartition_prise_8 | How are these plugs distributed? (8) | ${Outlet_Chase} = '8' |
| select_multiple sp3nh12 | Repartition_prise_9 | How are these plugs distributed? (9) | ${Outlet_Chase} = '9'. |
| select_multiple sp3nh12 | Repartition_prise_10 | How are these plugs distributed? (10) | ${Outlet_Chase} = '10' |
| select_one wr9js60 | Game_offered | Do you offer game? | |
| select_multiple vs1rc22 | Game_offered_context | In what context? | ${Game_offered} = 'yes'. |
| text | precisez_029 | specify | selected(${Game_offert_context}, 'other') |
| select_one aq1lk28 | Hunting_accompaniment | Are you accompanied on hunting trips? | |
| integer | Hunting_accompaniment_person_number | How many people? | ${Hunt_accompaniment} = 'yes'. |
| select_multiple du9jt17 | Hunting_accompaniment_name | Who's with you? | ${Hunt_accompaniment} = 'yes'. |
| text | precisez_030 | specify | selected(${Hunt_accompaniment_name}, 'other') |
| select_multiple zg5uv50 | Hunting_accompaniment_roles | What are the roles of your guides? | ${Hunt_accompaniment} = 'yes'. |
| text | precisez_031 | specify | selected(${Chasse_accompagnement_roles}, 'other') |

| | | | |
|---|---|---|---|
| select_one el26k55 | Dog_hunting | Do you hunt with dogs? | |
| text | Dog_hunting_roles | What are their roles? | ${Dog_Hunt} = 'yes'. |
| select_one wk5ym47 | Hunting_dogs_living_animals | Are dogs in contact with live animals? | ${Dog_Hunt} = 'yes'. |
| select_one fl9ud87 | Dog_hunting_products | Do dogs have access to hunting products? | ${Dog_Hunt} = 'yes'. |
| select_one kd2bq09 | Depecer_chiens_dechets | Do dogs have access to butchering waste? | ${Dog_Hunt} = 'yes'. |
| select_one ap6ph39 | Hunting_all_animals | Do you hunt all animals? | |
| select_multiple va5ws48 | Non-hunting_animals | Which ones don't you hunt? | ${All_Pets_Hunt} = 'no |
| text | precisez_032 | specify | selected(${Non-hunting_Animals}, 'other') |
| select_multiple gd9bg76 | Animals_not_hunting_rides | What are the reasons for this? | ${All_Pets_Hunt} = 'no |
| text | precisez_033 | specify | selected(${Animals_not_hunting_reasons}, 'other') |
| select_one nd0if48 | Youth_hunting_village | Do the children (< 18 years old) in your household hunt around the village/fields? | |
| select_multiple yp2cu14 | Teenagers_hunting_technical_village | What are the hunting techniques? | ${Chasse_adolescents_village} = 'oui' (yes) |
| text | precisez_034 | specify | selected(${Technical_village_teen_hunt}, 'other') |
| select_multiple ww4pi43 | Chasse_especes | What species are hunted? | ${Chasse_adolescents_village} = 'oui' (yes) |
| text | precisez_035 | specify | selected(${Species_Hunt}, 'other') |
| end_group | | | |
| begin_group | Game_transport | Game transport | selected(${Activities}, 'transport') |
| select_multiple zx8gp54 | Transport_animal_camping | How do you transport the hunted animal from the hunting ground to the camp? | ${Camp_Hunt} = 'yes |
| text | precisez_036 | specify | selected(${Transport_animal_camping}, 'other') |
| select_multiple wo43w44 | Transport_meat_village | How do you transport bushmeat from the forest to the village? | |
| text | precisez_037 | specify | selected(${Meat_transport_village}, 'other') |
| select_one od7le56 | Transport_protection_contact_game | Do you protect yourself from skin-to-skin contact with game? | |
| select_multiple ad3ih26 | Transport_protection_contact_game_how | How? | ${Transport_protection_contact_gibier} = 'oui' (yes) |
| text | precisez_038 | specify | selected(${Transport_protection_contact_game_comment}, 'other') |

| | | | |
|---|---|---|---|
| select_one kj48q30 | Transport_animaux_vivants | Do you ever transport live animals? | |
| select_multiple ww4pi43 | Transport_animaux_vivants_especes | Which ones? | ${Living_animals_transport} = 'yes'. |
| text | precisez_039 | specify | selected(${Living_animals_transport}, 'other') |
| select_multiple tj8nh86 | Transport_animaux_vivants_raisons | What are the reasons for this? | ${Living_animals_transport} = 'yes'. |
| text | precisez_040 | specify | selected(${Living_animal_transport_reasons}, 'other') |
| select_multiple kh52e08 | Transport_animaux_vivants_comment | How? | ${Living_animals_transport} = 'yes'. |
| text | precisez_041 | specify | selected(${Transport_living_animals_comment}, 'other') |
| select_one lb75v58 | Transport_gibier_toucher | During transport, can several game animals come into contact with each other? | |
| select_multiple ir8lh74 | Transport_gibier_toucher_raison | Why? | ${Transport_gibier_toucher} = 'no |
| text | precisez_042 | specify | selected(${Transport_gibier_toucher_raison}, 'autre') |
| select_multiple pv8lj28 | Transport_gibier_toucher_comment | How? | ${Transport_gibier_toucher} = 'no |
| text | precisez_043 | specify | selected(${Transport_gibier_toucher_comment}, 'other') |
| end_group | | | |
| begin_group | Depecer_gibier | Game butchering | selected(${Activities}, 'depecage') |
| select_multiple rs9co57 | Depecer_lieu | Where do you do the butchering? | |
| text | precisez_044 | specify | selected(${Depecer_lieu}, 'other') |
| select_one uk07t46 | Depecer_foret | Where in the forest? | selected(${Depecer_lieu}, 'foret') |
| text | precisez_045 | specify | ${Depecer_foret} = 'other |
| select_one px6vm84 | Depecer_village | Where in the village? | selected(${Depecer_lieu}, 'village') |
| text | precisez_046 | specify | ${Depecer_village} = 'other |
| select_one br4gd98 | Depecer_frequence_semaine | How many times a week do you cut up bushmeat? | |
| text | precisez_047 | specify | ${Depecer_frequence_semaine} = 'other |
| select_one lj2oc69 | Depecer_equipment | Do you wear any special equipment when butchering? | |
| select_multiple eb4zm22 | Remove_equipment_which | Which one? | ${Deploy_equipment} = 'yes |
| text | precisez_048 | specify | selected(${Depecer_equipement_lequel}, 'other') |
| select_multiple tm1zd18 | Depecer_surface | On which surface/material is the skinning performed? | |

| | | | |
|---|---|---|---|
| text | precisez_049 | specify | selected(${Depecer_surface}, 'other') |
| select_one cc63v52 | Depecer_surface_cleaning | Do you clean surfaces touched by fresh meat, blood or excrement? | |
| select_multiple mw2zl47 | Depecer_surface_cleaning_how | How? | ${Delete_cleaning_area} = 'yes |
| text | precisez_050 | specify | selected(${Depecer_surface_cleaning_comment}, 'other') |
| select_one lg0lj12 | Campement_proche_riviere | Is the hunting camp close to a running river? | ${Camp_Hunt} = 'yes |
| select_one or4ha23 | Depecer_injury | Have you ever been injured while butchering bushmeat? | |
| select_one fw3bi47 | Depecer_contact_fluids | Are you in contact with body fluids (e.g. blood, urine) and excrement during butchering? | |
| select_one ew9ea29 | Depecer_wash_hand_contact_meat | Are your hands washed after any contact with fresh meat? | |
| select_multiple pq7wy95 | Depecer_lavage_main_comment | How are they washed? | ${Depecer_lavage_main_contact_meat} = 'some_times' or ${Depecer_lavage_main_contact_meat} = 'always' |
| text | precisez_051 | specify | selected(${Depecer_lavage_main_comment}, 'other') |
| select_multiple ac1qt68 | Depecer_visceres_treatment | What do you do with the viscera? | |
| text | precisez_052 | specify | selected(${Depecer_visceres_treatment}, 'other') |
| select_multiple ba2vq86 | Depecer_visceres_lieu_jeter | Where do you throw it away? | selected(${Depecer_visceres_treatment}, 'throw') |
| text | precisez_053 | specify | selected(${Depecer_visceres_lieu_jeter}, 'other') |
| text | Depecer_visceres_destruction | How do you destroy them? | selected(${Depecer_visceres_processing}, 'destroy') |
| select_one zb2hv69 | Depecer_viande_fatiguee | Have you ever handled rotting bushmeat? | |
| select_multiple rv0kl10 | Depecer_viande_fatiguee_action | What do you do with this meat? | ${Depecer_viande_fatiguee} = 'oui' (yes) |
| text | precisez_054 | specify | selected(${Depecer_viande_fatiguee_action}, 'other') |
| select_multiple ba2vq86 | Depecer_viande_fatiguee_jeter_lieu | Where do you throw it away? | selected(${Depecer_viande_fatiguee_action}, 'jeter') |
| text | precisez_055 | specify | selected(${Depecer_viande_fatiguee_jeter_lieu}, 'other') |
| text | Depecer_meat_fatigue_destruction_how | How do you destroy it? | selected(${Depecer_viande_fatiguee_action}, 'detruire') |
| select_one kt85q53 | Depecer_chiens_proximite | Are any dogs near you during the butchering | |

| | | | |
|---|---|---|---|
| | | process? | |
| select_one pu1tz90 | Depecer_chiens_acces_dechets | Can dogs have access to butchering waste? | |
| end_group | | | |
| begin_group | Conservation | Conservation | selected(${Activities}, 'conservation') |
| select_multiple ez1ay79 | Conservation_produits_foret | Where do you keep the products of hunting in the forest? | ${Camp_Hunt} = 'yes |
| text | precisez_056 | specify | selected(${Conservation_products_foret}, 'other') |
| select_multiple ut7qj95 | Conservation_products_form_foret | In what form do you keep the products of hunting in the forest? | ${Camp_Hunt} = 'yes |
| text | precisez_057 | specify | selected(${Conservation_products_form_foret}, 'other') |
| select_one fh3nh71 | Conservation_foret_temps | For how long? | ${Camp_Hunt} = 'yes |
| select_multiple xp31d88 | Conservation_produits_village | Where do you keep the products of the village hunt? | |
| text | precisez_058 | specify | selected(${Conservation_village_products}, 'other') |
| select_multiple dc6jy89 | Village_form_products_conservation | In what form do you keep the products of the hunt in the village? | |
| text | precisez_059 | specify | selected(${Conservation_products_form_village}, 'other') |
| select_one mv0kk57 | Conservation_temps_village | For how long? | |
| end_group | | | |
| begin_group | Preparation_consumption | Preparation and consumption | selected(${Activities}, 'preparation') |
| select_one wa3di32 | Cutting_Injuries | Have you ever been injured while cutting bushmeat? | |
| select_one dx2cm78 | Parts_not_prepared | Are there any parts you don't prepare? | |
| select_multiple ep1dc77 | Parts_not_prepared_which | Which ones? | ${Parties_non_preparees} = 'oui' (yes) |
| text | precisez_060 | specify | selected(${Parties_not_prepared_which}, 'other') |
| select_multiple kx6re61 | Parts_not_prepared_treatment | What do you do with these unprepared parts? | ${Parties_non_preparees} = 'oui' (yes) |
| text | precisez_061 | specify | selected(${Non_prepared_treatment_parts}, 'other') |
| select_multiple zs3ak54 | Parts_not_prepared_dispose_place | Where do you dispose of them? | selected(${Parties_not_prepared_treatment}, 'throw') |
| text | precisez_062 | specify | selected(${Parties_not_prepared_jeter_endroit}, 'other') |
| text | Parts_not_prepared_destruction_how | How do you destroy it? | selected(${Parties_not_prepared_treatment}, 'detruire') |

| | | | |
|---|---|---|---|
| select_multiple ag4fj33 | Reception_status_products | In what condition do you receive your products? | |
| text | precisez_063 | specify | selected(${Reception_status_products}, 'other') |
| select_one wb9oc48 | Cuisine_frequence_semaine | How often do you prepare bushmeat per week? | |
| text | precisez_064 | specify | ${Cuisine_frequence_semaine} = 'other |
| select_one ep66d73 | Preparation_wash_hand_contact_fresh_meat | Are your hands washed after any contact with fresh meat? | |
| select_multiple ve9yd88 | Preparation_wash_hand_contact_fresh_meat_how | How are they washed? | ${Preparation_lavage_main_contact_viandefraiche} = 'some_times' or ${Preparation_lavage_main_contact_viandefraiche} = 'always' |
| text | precisez_065 | specify | selected(${Preparation_wash_hand_contact_fresh_meat_comment}, 'other') |
| select_multiple yy0nz46 | Preparation_fatigue_meat_treatment | What do you do with tired bushmeat (decomposition process started)? | |
| text | precisez_066 | specify | selected(${Preparation_meat_fatiguee_treatment}, 'other') |
| select_multiple zs3ak54 | Preparation_fatigue_meat_throw_place | Where do you dispose of them? | selected(${Preparation_meat_fatiguee_treatment}, 'throw') |
| text | Precise_067 | Specify | selected(${Preparation_meat_fatiguee_jeter_lieu}, 'other') |
| text | Preparation_fatigue_meat_destruction_how | How do you destroy it? | selected(${Preparation_meat_fatiguee_treatment}, 'detruire') |
| select_multiple sn8uq51 | Cuisine_viande_comment | How do you cook bushmeat? | |
| text | precisez_068 | specify | selected(${Meat_kitchen_comment}, 'other') |
| end_group | | | |
| begin_group | Pets | Pets | |
| select_one jf5uq47 | Pets_ownership | Have you had pets in the last 12 months? | |
| select_multiple qx5oo16 | Pets_ownership_which | Which ones? | ${Domestic_Pets_Property} = 'yes |
| text | precisez_069 | specify | selected(${domestic_animals_ownership_which}, 'other') |
| select_one oc2sy29 | Acces_produits_chiens_chasse | Do the dog(s) have access to the products of the hunt? | selected(${domestic_animals_ownership_which}, 'dog') |
| select_one xy4je85 | Acces_produits_chiens_dechets | Do the dog(s) have access to kitchen and/or meal waste? | selected(${domestic_animals_ownership_which}, 'dog') |

| | | | |
|---|---|---|---|
| select_one fv15k54 | Acces_produits_chats_chasse | Do the cat(s) have access to the products of the hunt? | selected(${domestic_animals_ownership_which}, 'cat') |
| select_one ka85o11 | Acces_produits_chats_dechets | Do the cat(s) have access to kitchen and/or meal waste? | selected(${domestic_animals_ownership_which}, 'cat') |
| select_one df9nx50 | Acces_produits_porcs_chasse | Do the pig(s) have access to hunting products? | selected(${domestic_animals_ownership_which}, 'pig') |
| select_one cy3ld97 | Acces_porc_waste_products | Do the pig(s) have access to kitchen and/or meal waste? | selected(${domestic_animals_ownership_which}, 'pig') |
| select_one nu0br90 | Acces_produits_animauxelevage_chasse | Do farm animals have access to hunting products? | selected(${Domestic_Animals_propriete_lesquels}, 'goat') or selected(${Domestic_Animals_propriete_lesquels}, 'sheep') or selected(${Domestic_Animals_propriete_lesquels}, 'cattle') or selected(${Domestic_Animals_propriete_lesquels}, 'chicken') |
| select_one nu0br90 | Access_animal_products_farming_waste | Do farm animals have access to kitchen and/or meal waste? | selected(${Domestic_Animals_propriete_lesquels}, 'goat') or selected(${Domestic_Animals_propriete_lesquels}, 'sheep') or selected(${Domestic_Animals_propriete_lesquels}, 'cattle') or selected(${Domestic_Animals_propriete_lesquels}, 'chicken') |
| end_group | | | |
| begin_group | Risk_perception | Perception of risk/danger | |
| select_one ez6pm81 | Contact_animalvivant | Have you been in physical contact with a live animal in the last 12 months? | |
| select_multiple me5zf83 | Contact_animalvivant_especies | With animals of what species? | ${Living_Pet_Contact} = 'yes'. |
| text | precisez_070 | specify | selected(${Contact_animal_live}, 'other') |
| select_one qu8xx12 | Animal_bite | Have you been bitten by an animal in the last 12 months? | |
| select_multiple yf5hm98 | Bite_animal_circumstance | Under what circumstances? | ${Animal_bite} = 'one_two_times' or ${Animal_bite} = 'three_five_times' or ${Animal_bite} = 'more_five_times'. |
| text | precisez_071 | specify | selected(${Animal_bite_circumstance}, 'other') |

| | | | |
|---|---|---|---|
| select_multiple me5zf83 | Bite_animal_species | With animals of what species? | ${Animal_bite} = 'one_two_times' or ${Animal_bite} = 'three_five_times' or ${Animal_bite} = 'more_five_times'. |
| text | precisez_072 | specify | selected(${Animal_bite_species}, 'other') |
| select_one ju8ne58 | Animal_claw | Have you been scratched by an animal in the last 12 months? | |
| select_multiple ji1ze29 | Claw_animal_circumstance | Under what circumstances? | ${Griffe_animal} = 'une_deux_fois' or ${Griffe_animal} = 'trois_cinq_fois' or ${Griffe_animal} = 'plus_cinq_fois'. |
| text | precisez_073 | specify | selected(${Griffe_animal_circonstance}, 'other') |
| select_multiple me5zf83 | Animal_species_claw | By animals of what species? | ${Griffe_animal} = 'une_deux_fois' or ${Griffe_animal} = 'trois_cinq_fois' or ${Griffe_animal} = 'plus_cinq_fois'. |
| text | precisez_074 | specify | selected(${Griffe_animal_espece}, 'other') |
| select_one xe96n92 | Product_normal_signs | Over the past 12 months, have you come across any abnormal signs on hunting products? | |
| integer | Signs_anormal_products_frequency | How many times has this happened to you? | ${Product_normal_signs} = 'no |
| select_multiple li8if61 | Abnormal_signs_of_specified_products | With what species of animal? | ${Product_normal_signs} = 'no |
| text | precisez_075 | specify | selected(${Signs_anormal_products_especes}, 'other') |
| select_multiple ho7ug89 | abnormal_signs_products_treatment | What did you do with the product in question? | ${Product_normal_signs} = 'no |
| text | precisez_076 | specify | selected(${Anomalous_signs_treatment_products}, 'other') |
| select_multiple zs3ak54 | Abnormal_signs_products_throw_place | Where do you dispose of them? | selected(${Signs_abnormal_treatment_products}, 'throw') |
| text | precisez_077 | specify | selected(${Anomalous_signs_products_throw_place}, 'other') |
| text | Abnormal_signs_destruction_products_how | How do you destroy it? | selected(${Anomalous_signs_treatment_products}, 'detruire') |
| select_one | Abnormal_signs_produ | Have you told anyone about | ${Product_normal_signs} = |

| | | | |
|---|---|---|---|
| yi28g08 | cts_to_come | this discovery? | 'no |
| select_multiple un06u98 | Signs_of_anormal_products_which | Who? | ${Annormal_signs_products} = 'yes |
| text | precisez_078 | specify | selected(${Signs_anormal_products_prevenir_qui}, 'other') |
| select_one bc6eh68 | Find_dead_wild_animal | In the past 12 months, have you come across a dead wild animal? | |
| integer | Find_wild_animal_death_frequency | How many times has this happened to you? | ${Find_wild_dead_animal} = 'yes'. |
| select_multiple li8if61 | Trouver_animal_sauvage_mort_espece | With what species of animal? | ${Find_wild_dead_animal} = 'yes'. |
| text | precisez_079 | specify | selected(${Find_wild_dead_animal}, 'other') |
| select_multiple nh9de12 | Find_wild_animal_death_action | What did you do? | ${Find_wild_dead_animal} = 'yes'. |
| text | precisez_080 | specify | selected(${Find_wild_animal_death_action}, 'other') |
| select_one rp8me13 | Trouver_animal_sauvage_mort_prevenir | Have you told anyone about this discovery? | ${Find_wild_dead_animal} = 'yes'. |
| select_multiple vf5ez25 | Trouver_animal_sauvage_mort_prevenir_qui | Who? | ${Find_wild_dead_animal} = 'yes'. |
| text | precisez_081 | specify | selected(${Find_wild_dead_animal_prevenir_who}, 'other') |
| select_one rt6ep37 | Find_dead_domestic_animal | In the past 12 months, have you come across a dead pet? | |
| integer | Find_domestic_animal_death_frequency | How many times has this happened to you? | ${Find_domestic_animal_dead} = 'yes'. |
| select_multiple rd7yw62 | Find_domestic_animal_death_species | With what species of animal? | ${Find_domestic_animal_dead} = 'yes'. |
| text | precisez_082 | specify | selected(${Find_domestic_dead_animal}, 'other') |
| select_multiple bq3pw07 | Find_domestic_animal_death_action | What did you do? | ${Find_domestic_animal_dead} = 'yes'. |
| text | precisez_083 | specify | selected(${Find_domestic_animal_death_action}, 'other') |
| select_one pd4rh18 | Trouver_animal_domestic_mort_prevenir | Have you told anyone about these discoveries? | ${Find_domestic_animal_dead} = 'yes'. |
| select_multiple ie6te29 | Trouver_animal_domestic_mort_prevenir_qui | Who? | ${Find_domestic_dead_animal} = 'yes'. |
| text | precisez_084 | specify | selected(${Trouver_animal_domestique_mort_prevenir_qui}, 'other') |
| select_one rq9ny36 | Hunting_animal_illness_signs | In the past 12 months, have you come across any signs of illness in the animals you hunt? | |
| integer | Signs_of_animal_illness_hunting_frequency | How many times has this happened to you? | ${Hunting_animal_illness_signs} = 'yes'. |

| | | | |
|---|---|---|---|
| select_multiple li8if61 | Signs_of_animal_illnesses_hunting_species | With what species of animal? | ${Hunting_animal_illness_signs} = 'yes'. |
| text | precisez_085 | specify | selected(${Hunting_animal_illness_signs}, 'other') |
| select_multiple vz1cz24 | Signs_of_animal_illness_hunting_nature | What are these signs? | ${Hunting_animal_illness_signs} = 'yes'. |
| text | precisez_086 | specify | selected(${Animal_Disease_Signs_Hunting_Nature}, 'other') |
| select_multiple sk5nq39 | Signs_of_animal_illness_hunting_action | What did you do? | ${Hunting_animal_illness_signs} = 'yes'. |
| text | precisez_087 | specify | selected(${Animal_illness_signs_hunting_action}, 'other') |
| select_one pf5kg71 | Signs_of_animal_illness_hunting_future | Have you told anyone about this discovery? | ${Hunting_animal_illness_signs} = 'yes'. |
| select_multiple rq0ut60 | Signs_of_animal_illness_hunting_prevent_who | Who? | ${Preventable_animal_illness_signs} = 'yes'. |
| text | precisez_088 | specify | selected(${Animal_illness_signs_hunting_prevent_who}, 'other') |
| select_one gw3vu83 | Domestic_animal_illness_signs | Over the past 12 months, have you come across any signs of illness in the pets you keep? | |
| integer | Signs_of_domestic_animal_illness_count | How many animals were affected? | ${Pet_Disease_Signs} = 'yes'. |
| select_multiple ae5dj10 | Signs of domestic_animal_disease | With what species of animal? | ${Pet_Disease_Signs} = 'yes'. |
| text | precisez_089 | specify | selected(${Domestic_animal_illness_signs}, 'other') |
| select_multiple vn1ap87 | Signs_of_domestic_animal_illness_action | What did you do? | ${Pet_Disease_Signs} = 'yes'. |
| text | precisez_090 | specify | selected(${Domestic_animal_illness_signs_action}, 'other') |
| select_one ec5jk61 | Signs_of_domestic_animal_disease_future | Have you told anyone about this discovery? | ${Pet_Disease_Signs} = 'yes'. |
| select_multiple el45z22 | Signs_of_domestic_animal_illness_which_will_occur | Who? | ${Domestic_animal_illness_signs} = 'yes'. |
| text | precisez_091 | specify | selected(${Domestic_animal_illness_signs_for_who}, 'other') |
| begin_group | Risques_chasse_manipulation | Can you list three major risks associated with hunting or handling bushmeat? | |
| text | Risques_chasse_manipulation_1 | Risk 1: | |

| | | | |
|---|---|---|---|
| text | Risques_chasse_manipulation_2 | Risk 2: | |
| text | Risques_chasse_manipulation_3 | Risk 3: | |
| end_group | | | |
| select_one iq9zk31 | Animals_wild_care | Do you believe that wild animals can cure disease? | |
| select_multiple li8if61 | Animals_sauvages_soigner_especes | What kind of animals? | ${Wild_Pets_Treat} = 'yes |
| text | precisez_092 | specify | selected(${wild_animals_care_species}, 'other') |
| text | Wild_animals_healing_illnesses | Against what? | ${Wild_Pets_Treat} = 'yes |
| select_one zq3la00 | Transmission_maladies_animaux | Do you think animals can transmit diseases to humans? | |
| select_one fn4je65 | Transmission_maladies_animaux_connaissance | Do you know of any diseases that can be transmitted from animals to humans? | ${Pet_Disease_Transmission} = 'yes'. |
| begin_group | Transmission_maladies_animaux_citer | Can you name two diseases? | ${Pet_disease_transmission_knowledge} = 'yes'. |
| text | Transmission_maladies_animaux_citer_1 | Disease 1 : | |
| text | Transmission_maladies_animaux_citer_2 | Disease 2: | |
| end_group | | | |
| select_multiple li8if61 | Transmission_maladies_animaux_especes | Can you list the animal species that transmit these diseases? | ${Pet_disease_transmission_knowledge} = 'yes'. |
| text | precisez_093 | specify | selected(${Pet_Disease_Transmission}, 'other') |
| select_one am9vk61 | Transmission_of_animal_illnesses_to_sick_people | Do you know anyone who has contracted an illness or health problem through contact with animals (handling and/or consumption)? | ${Pet_Disease_Transmission} = 'yes'. |
| select_multiple sd0pf73 | Contacts_risque_humain_animal | Of all the different types of contact between animals and humans, which do you think is the most risky? | |
| select_one or1tw70 | Disease_protection_measures | Can you list two measures to protect against animal-borne diseases? | ${Pet_Disease_Transmission} = 'yes'. |
| begin_group | Mesures_protection_maladies_citer | Measures | ${Disease_protection_measures} = 'yes'. |
| text | Mesures_protection_maladies_citer_1 | Measure 1: | |
| text | Mesures_protection_maladies_citer_2 | Measure 2: | |

| | | | |
|---|---|---|---|
| end_group | | | |
| select_multiple ac5bt15 | Measures_for_protecting_applied_illnesses | Do you apply any of these measures? | ${Disease_protection_measures} = 'yes'. |
| end_group | | | |
| text | Comment | General comments | |
| select_one mu3xg98 | Entretien_ulterieur | Would you be willing to take part in the same interview at a later date? | |
| text | Telephone | Phone number | ${Litter_Maintenance} = 'yes'. |

**Table 1. Options associated with each multiple-choice question**

| List name | name | label |
|---|---|---|
| cs5fx91 | natacha | Natacha Efoua Tomo |
| cs5fx91 | other | Other |
| qw1pg64 | ogooue_lolo | Ogooué Lolo |
| qw1pg64 | other | Other |
| sz6zl15 | mulundu | Mulundu |
| sz6zl15 | other | Other |
| tu6wg63 | bembicani | BEMBICANI |
| tu6wg63 | malende | MALENDE |
| tu6wg63 | boundzoumba | BOUNDZOUMBA |
| tu6wg63 | lipaka_2 | LIPAKA 2 |
| tu6wg63 | kessipoughou | KESSIPOUGHOU |
| tu6wg63 | bakoussou_ndekabalandji | BAKOUSSOU-NDEKABALANDJI |
| tu6wg63 | baposso | BAPOSSO |
| tu6wg63 | boundzi | BOUNDZI |
| tu6wg63 | nzela | NZELA |
| tu6wg63 | kokomounguele | KOKOMOUNGUELE |
| tu6wg63 | mana_mana | MANA-MANA |
| tu6wg63 | other | Other |
| vb0bw83 | bembicani | Bembicani |
| vb0bw83 | lipaka_1 | Lipaka1 |
| vb0bw83 | layemba | Layemba |
| vb0bw83 | nianianguina | Nianianguina |
| vb0bw83 | poungui | Poungui |
| vb0bw83 | other | Other |
| oc52s06 | malende | Malende |
| oc52s06 | mikouyi | Mikouyi |
| oc52s06 | other | Other |
| ef2hh35 | nzondet | Nzondet |
| ef2hh35 | moumba | Moumba |
| ef2hh35 | boundjoumba | Boundjoumba |
| ef2hh35 | other | Other |

| | | |
|---|---|---|
| pm6xo35 | lekeni_moulessi | Lekeni_moulessi |
| pm6xo35 | lipaka_2 | Lipaka2 |
| pm6xo35 | youlandzambi | Youlandzambi |
| pm6xo35 | other | Other |
| bp9sl17 | kessipoughou | Kessipoughou |
| bp9sl17 | other | Other |
| al8mf60 | bakoussou | Bakoussou |
| al8mf60 | ndekabalandji | Ndekabalandji |
| al8mf60 | mekouka | Mekouka |
| al8mf60 | siamangwandza | Siamangwandza |
| al8mf60 | other | Other |
| eg3qv46 | baposso | Baposso |
| eg3qv46 | other | Other |
| xh62m65 | boundzi | Boundzi |
| xh62m65 | other | Other |
| cf2dr77 | dibangagnia | Dibangagnia |
| cf2dr77 | lipopa | Lipopa |
| cf2dr77 | makougoulou | Makougoulou |
| cf2dr77 | mikouyi | Mikouyi |
| cf2dr77 | nzela | Nzela |
| cf2dr77 | other | Other |
| pc1sf31 | kokomounguele | Kokomounguele |
| pc1sf31 | other | Other |
| lc7tt73 | mana_mana | Mana-Mana |
| lc7tt73 | other | Other |
| zp92d01 | m | M |
| zp92d01 | f | F |
| py01n08 | gabonaise | Gabonese |
| py01n08 | other | Other |
| qq07e03 | awandji | Awandji |
| qq07e03 | babongo | Babongo |
| qq07e03 | kota | Kota |
| qq07e03 | sake | Sake |
| qq07e03 | adouma | Adouma |
| qq07e03 | other | Other |
| pc8fd16 | survey_place | Survey location |
| pc8fd16 | other | Other |
| ta21o25 | marie | Married |
| ta21o25 | single | Single |
| ta21o25 | widower_widow | Widow |
| ta21o25 | other | Other |
| cj4yc09 | yes | yes |
| cj4yc09 | no | no |
| zh5ym19 | no | No |
| zh5ym19 | primary | Primary |
| zh5ym19 | secondary | Secondary |

| | | |
|---|---|---|
| zh5ym19 | university | University |
| zh5ym19 | other | Other |
| mo75z76 | hunting | Hunting |
| mo75z76 | transport | Game transportation |
| mo75z76 | cleaning | Game butchering |
| mo75z76 | conservation | Game preservation |
| mo75z76 | preparation | Preparing game |
| dz9jk21 | yes | Yes |
| dz9jk21 | no | No |
| nf7mv96 | agriculture | Agriculture |
| nf7mv96 | fishing | Fishing |
| nf7mv96 | other | Other |
| hg7rt38 | eat | To feed yourself |
| hg7rt38 | sell | To sell |
| hg7rt38 | ceremonies | For traditional ceremonies |
| hg7rt38 | treat | To treat diseases |
| hg7rt38 | other | Other |
| gb6ts02 | villagers | Other villagers |
| gb6ts02 | restaurateurs | Restaurateurs you know |
| gb6ts02 | passers-by | To passers-by who stop on the road |
| gb6ts02 | other | Other |
| gh8ou58 | main | Main source of income |
| gh8ou58 | secondary | Secondary source of income |
| ww4pi43 | cephalophes | duiker |
| ww4pi43 | potamocheres | Bushpig |
| ww4pi43 | pigs_epics | Porcupines |
| ww4pi43 | little_singes | Little monkeys |
| ww4pi43 | big_singes | Great apes (gorillas, chimpanzees) |
| ww4pi43 | bat_souris | Bats |
| ww4pi43 | pangolin | Pangolin |
| ww4pi43 | rat_palmiste | Palmetto rat |
| ww4pi43 | fox | Fox |
| ww4pi43 | herisson | Hedgehog |
| ww4pi43 | viper | Vipers |
| ww4pi43 | reptiles | Reptiles |
| ww4pi43 | toucan | Toucan |
| ww4pi43 | guinea fowl | Guinea fowl |
| ww4pi43 | chat_tigre | Tiger cat |
| ww4pi43 | cat_huant | Booing cat |
| ww4pi43 | chevrotin | Chevrotin |
| ww4pi43 | civet | Civet |
| ww4pi43 | crocodile | Crocodile |
| ww4pi43 | caiman | Caiman |
| ww4pi43 | pithon | Pithon |
| ww4pi43 | squirrel | Squirrel |
| ww4pi43 | armadillo | Armadillo |

| | | |
|---|---|---|
| ww4pi43 | elephant | Elephant |
| ww4pi43 | buffalo | Buffalo |
| ww4pi43 | other | Other |
| ia2gk86 | yes | Yes |
| ia2gk86 | no | No |
| pr3sf30 | opening | Hunting season |
| pr3sf30 | ceremonies | Ceremonies |
| pr3sf30 | rainy_season | Rainy season |
| pr3sf30 | dry_season | Dry season |
| pr3sf30 | traditional | Traditional rites |
| pr3sf30 | other | Other |
| qo1lb58 | rifle | Hunting with a rifle |
| qo1lb58 | trap | Trapping |
| qo1lb58 | net | Net hunting |
| qo1lb58 | other | Other |
| au3ky88 | rifle | With a shotgun blast |
| au3ky88 | machete | With a machete |
| au3ky88 | living | Live capture |
| au3ky88 | stick | With a stick |
| au3ky88 | other | Other |
| vy0ic01 | village | Near / Next to the village |
| vy0ic01 | fields | Around the fields |
| vy0ic01 | drill | In the forest |
| vy0ic01 | other | Other |
| np6ge53 | day | The day |
| np6ge53 | night | The night |
| ay35j48 | <1 | less than 1 time |
| ay35j48 | 1_2 | 1-2 |
| ay35j48 | 2_4 | 2-4 |
| ay35j48 | 5_8 | 5-8 |
| ay35j48 | >8 | >8 |
| bq7gm71 | less_one_day | Less than a day |
| bq7gm71 | one_two_days | One to two days |
| bq7gm71 | more_two_days | More than two days |
| gf7rz42 | yes | Yes |
| gf7rz42 | no | No |
| an8zq70 | 1 | 1 |
| an8zq70 | 2 | 2 |
| an8zq70 | 3 | 3 |
| an8zq70 | 4 | 4 |
| an8zq70 | 5 | 5 |
| an8zq70 | 6 | 6 |
| an8zq70 | 7 | 7 |
| an8zq70 | 8 | 8 |
| an8zq70 | 9 | 9 |
| an8zq70 | 10 | 10 |

| | | |
|---|---|---|
| sp3nh12 | vend_plus_moitie_consomme_reste | I give or sell more than half and consume the rest |
| sp3nh12 | consomme_plus_moitie_vend_reste | I consume more than half and sell/donate the rest |
| sp3nh12 | sell_all | I sell everything |
| sp3nh12 | consume_all | I consume everything |
| sp3nh12 | share_owner_carrier_rifle | I share with a gun owner and/or carrier |
| wr9js60 | yes | Yes |
| wr9js60 | no | No |
| vs1rc22 | family | Close family with whom it is customary to share |
| vs1rc22 | barter | Barter for another good or service |
| vs1rc22 | fete | Party/Ceremony |
| vs1rc22 | other | Other |
| aq1lk28 | yes | Yes |
| aq1lk28 | no | No |
| du9jt17 | woman | Your wife |
| du9jt17 | children | Your children |
| du9jt17 | hunters | Other hunters |
| du9jt17 | young people | Village youth (<18 years) |
| du9jt17 | other | Other |
| zg5uv50 | hunting | Hunting |
| zg5uv50 | set up | Setting up camp |
| zg5uv50 | food | Preparing food |
| zg5uv50 | transport | Transporting hunting products |
| zg5uv50 | smoking | Smoking bushmeat |
| zg5uv50 | other | Other |
| el26k55 | yes | Yes |
| el26k55 | no | No |
| wk5ym47 | yes | Yes |
| wk5ym47 | no | No |
| fl9ud87 | yes | Yes |
| fl9ud87 | no | No |
| kd2bq09 | yes | Yes |
| kd2bq09 | no | No |
| ap6ph39 | yes | Yes |
| ap6ph39 | no | No |
| va5ws48 | turtle | Turtle |
| va5ws48 | snake | Snake |
| va5ws48 | panther | Panther |
| va5ws48 | gorilla | Gorilla |
| va5ws48 | other | Other |
| gd9bg76 | totem | Totem |
| gd9bg76 | don't_want_to_hunt | Don't want to hunt |
| gd9bg76 | prohibited_hunting | Species prohibited from hunting |
| gd9bg76 | betes_feroces | Fierce beasts |

| | | |
|---|---|---|
| gd9bg76 | other | Other |
| nd0if48 | yes | Yes |
| nd0if48 | no | No |
| yp2cu14 | trap | Traps (snares) |
| yp2cu14 | lance_pierre | Slingshot |
| yp2cu14 | dog | Dog |
| yp2cu14 | other | Other |
| zx8gp54 | without_equipment | No special equipment |
| zx8gp54 | sachers | In the bags |
| zx8gp54 | hoods | In traditional baskets/Hoots |
| zx8gp54 | bowls | In bowls |
| zx8gp54 | bags_riz | Rice bags |
| zx8gp54 | other | Other |
| wo43w44 | without_equipment | No special equipment |
| wo43w44 | plastic_bags | In the bags |
| wo43w44 | hood | In traditional baskets/hoots |
| wo43w44 | bowls | In bowls |
| wo43w44 | bags_riz | Rice bags |
| wo43w44 | other | Other |
| od7le56 | yes | Yes |
| od7le56 | no | No |
| ad3ih26 | plastic_protection | Plastic protection |
| ad3ih26 | protection_hotte | Protection with hood |
| ad3ih26 | other | Other |
| kj48q30 | yes | Yes |
| kj48q30 | no | No |
| tj8nh86 | ceremonies | Ceremonies |
| tj8nh86 | clients_musulmans | Muslim customers |
| tj8nh86 | other | Other |
| kh52e08 | without_equipment | No special equipment |
| kh52e08 | bags | In the bags |
| kh52e08 | hoods | In traditional baskets/hoots |
| kh52e08 | bowls | In bowls |
| kh52e08 | bags_riz | In rice bags |
| kh52e08 | other | Other |
| lb75v58 | yes | Yes |
| lb75v58 | no | No |
| ir8lh74 | tired_meat | Separated tired meat |
| ir8lh74 | other | Other |
| pv8lj28 | bags | Sachets |
| pv8lj28 | other | Other |
| rs9co57 | drill | In the forest |
| rs9co57 | village | In the village |
| rs9co57 | other | Other |
| uk07t46 | au_campement | At the camp |
| uk07t46 | other | Other |

| | | |
|---|---|---|
| px6vm84 | around_the_house | Around the house |
| px6vm84 | in_the_house | In the house |
| px6vm84 | other | Other |
| br4gd98 | more_than_twice_weekly | Less than once a week |
| br4gd98 | twice_weekly | Once a week |
| br4gd98 | once_a_week | Twice a week |
| br4gd98 | less_than_once_week | More than twice a week |
| br4gd98 | other | Other |
| lj2oc69 | yes | Yes |
| lj2oc69 | no | No |
| eb4zm22 | gloves | Gloves |
| eb4zm22 | mask | Mask |
| eb4zm22 | apron | Apron |
| eb4zm22 | other | Other |
| tm1zd18 | par_terre | On the ground |
| tm1zd18 | sur_table | On a table |
| tm1zd18 | tole | On sheet metal |
| tm1zd18 | other | Other |
| cc63v52 | yes | Yes |
| cc63v52 | no | No |
| mw2zl47 | water_simple | Wash with plain water |
| mw2zl47 | detergent | Wash with detergent |
| mw2zl47 | soap | Wash with soap |
| mw2zl47 | other | Other |
| lg0lj12 | yes | Yes |
| lg0lj12 | no | No |
| or4ha23 | never | Never |
| or4ha23 | one_time_year | Once a year |
| or4ha23 | more_once_year | More than once a year |
| fw3bi47 | never | Never |
| fw3bi47 | some_times | A few times |
| fw3bi47 | always | Always |
| ew9ea29 | never | Never |
| ew9ea29 | some_times | A few times |
| ew9ea29 | always | Always |
| pq7wy95 | water_simple | Plain water |
| pq7wy95 | soap | With soap |
| pq7wy95 | detergent | With detergent |
| pq7wy95 | other | Other |
| ac1qt68 | throw | Discard |
| ac1qt68 | destroy | Destroy |
| ac1qt68 | bury | Bury |
| ac1qt68 | consume | Keep for consumption |
| ac1qt68 | sell | Sell |
| ac1qt68 | keep | Keep |
| ac1qt68 | other | Other |

| | | |
|---|---|---|
| ba2vq86 | camp | In the camp |
| ba2vq86 | village | In the village |
| ba2vq86 | drill | In the forest |
| ba2vq86 | water_point | In watering holes |
| ba2vq86 | other | Other |
| zb2hv69 | yes | Yes |
| zb2hv69 | no | No |
| rv0kl10 | consume | Consuming with the family |
| rv0kl10 | sell | Sell |
| rv0kl10 | share | Share with others |
| rv0kl10 | throw | Discard |
| rv0kl10 | destroy | Destroy |
| rv0kl10 | bury | Bury |
| rv0kl10 | other | Other |
| kt85q53 | yes | Yes |
| kt85q53 | no | No |
| pu1tz90 | yes | Yes |
| pu1tz90 | no | No |
| ez1ay79 | drill | In the open air |
| ez1ay79 | village | In a fenced-in area |
| ez1ay79 | riviere | In the river |
| ez1ay79 | other | Other |
| ut7qj95 | fresh | Fresh |
| ut7qj95 | smoke | Goat/Smoke |
| ut7qj95 | other | Other |
| fh3nh71 | days | A few days |
| fh3nh71 | weeks | A few weeks |
| fh3nh71 | month | A few months |
| fh3nh71 | more_one_year | More than a year |
| xp31d88 | air_libre | In the open air |
| xp31d88 | place_clot | In a fenced-in area |
| xp31d88 | refrigerator | In a refrigerator |
| xp31d88 | freezer | In a freezer |
| xp31d88 | other | Other |
| dc6jy89 | fresh | Fresh |
| dc6jy89 | smoke | Goat/Smoke |
| dc6jy89 | other | Other |
| mv0kk57 | days | A few days |
| mv0kk57 | weeks | A few weeks |
| mv0kk57 | month | A few months |
| mv0kk57 | more_one_year | More than a year |
| wa3di32 | never | Never |
| wa3di32 | once_once | Only once |
| wa3di32 | several | Several times |
| dx2cm78 | yes | Yes |
| dx2cm78 | no | No |

| | | |
|---|---|---|
| ep1dc77 | skin | Skin |
| ep1dc77 | other | Other |
| kx6re61 | throw | Discard |
| kx6re61 | destroy | Destroy |
| kx6re61 | bury | Bury |
| kx6re61 | consume | Keep for consumption |
| kx6re61 | sell | Sell |
| kx6re61 | keep | Keep |
| kx6re61 | other | Other |
| zs3ak54 | village | In the village |
| zs3ak54 | drill | In the forest |
| zs3ak54 | water_points | In watering holes |
| zs3ak54 | other | Other |
| ag4fj33 | fees | Fees |
| ag4fj33 | smoke | Smoke |
| ag4fj33 | fatigue | Tired |
| ag4fj33 | other | Other |
| wb9oc48 | more_than_twice_weekly | Less than once a week |
| wb9oc48 | twice_weekly | Once a week |
| wb9oc48 | once_a_week | Twice a week |
| wb9oc48 | less_than_once_week | More than twice a week |
| wb9oc48 | other | Other |
| ep66d73 | never | Never |
| ep66d73 | some_times | A few times |
| ep66d73 | always | Always |
| ve9yd88 | water_simple | Plain water |
| ve9yd88 | soap | With soap |
| ve9yd88 | detergent | With detergent |
| ve9yd88 | other | Other |
| yy0nz46 | throw | Discard |
| yy0nz46 | destroy | Destroy |
| yy0nz46 | enter | Bury |
| yy0nz46 | consume | Keep for consumption |
| yy0nz46 | sell | Sell |
| yy0nz46 | keep | Keep |
| yy0nz46 | other | Other |
| sn8uq51 | fire | Wood-fired |
| sn8uq51 | gas | Gas |
| sn8uq51 | other | Other |
| jf5uq47 | yes | Yes |
| jf5uq47 | no | No |
| qx5oo16 | dog | Dog |
| qx5oo16 | cat | Cat |
| qx5oo16 | goat | Goat |
| qx5oo16 | sheep | Sheep |
| qx5oo16 | cattle | Cattle |

| | | |
|---|---|---|
| qx5oo16 | pork | Pork |
| qx5oo16 | chicken | Chicken |
| qx5oo16 | other | Other |
| oc2sy29 | yes | Yes |
| oc2sy29 | no | No |
| xy4je85 | yes | Yes |
| xy4je85 | no | No |
| fv15k54 | yes | Yes |
| fv15k54 | no | No |
| ka85o11 | yes | Yes |
| ka85o11 | no | No |
| df9nx50 | yes | Yes |
| df9nx50 | no | No |
| cy3ld97 | yes | Yes |
| cy3ld97 | no | No |
| nu0br90 | yes | Yes |
| nu0br90 | no | No |
| ez6pm81 | yes | Yes |
| ez6pm81 | no | No |
| me5zf83 | cephalophes | duiker |
| me5zf83 | potamocheres | Bushpig |
| me5zf83 | pigs_epics | Porcupines |
| me5zf83 | little_singes | Little monkeys |
| me5zf83 | big_singes | Great apes (gorillas, chimpanzees) |
| me5zf83 | bat_souris | Bats |
| me5zf83 | pangolin | Pangolin |
| me5zf83 | rat_palmiste | Palmetto rat |
| me5zf83 | fox | Fox |
| me5zf83 | herisson | Hedgehog |
| me5zf83 | viper | Vipers |
| me5zf83 | reptiles | Reptiles |
| me5zf83 | toucan | Toucan |
| me5zf83 | guinea fowl | Guinea fowl |
| me5zf83 | chat_tigre | Tiger cat |
| me5zf83 | cat_huant | Booing cat |
| me5zf83 | chevrotin | Chevrotin |
| me5zf83 | civet | Civet |
| me5zf83 | crocodile | Crocodile |
| me5zf83 | caiman | Caiman |
| me5zf83 | pithon | Pithon |
| me5zf83 | squirrel | Squirrel |
| me5zf83 | armadillo | Armadillo |
| me5zf83 | elephant | Elephant |
| me5zf83 | buffalo | Buffalo |
| me5zf83 | dog | Dog |
| me5zf83 | cat | Cat |

| | | |
|---|---|---|
| me5zf83 | goat | Goat |
| me5zf83 | sheep | Sheep |
| me5zf83 | cattle | Cattle |
| me5zf83 | pork | Pork |
| me5zf83 | chicken | Chicken |
| me5zf83 | other | Other |
| qu8xx12 | never | Never |
| qu8xx12 | once_twice | Once or twice |
| qu8xx12 | three_five_times | Three to five times |
| qu8xx12 | more_five_times | More than five times |
| yf5hm98 | trap | In the trap |
| yf5hm98 | drill | Meeting in the forest |
| yf5hm98 | village | In the village |
| yf5hm98 | other | Other |
| ju8ne58 | never | Never |
| ju8ne58 | once_twice | Once or twice |
| ju8ne58 | three_five_times | Three to five times |
| ju8ne58 | more_five_times | More than five times |
| ji1ze29 | trap | In the trap |
| ji1ze29 | drill | Meeting in the forest |
| ji1ze29 | village | In the village |
| ji1ze29 | other | Other |
| xe96n92 | no | Yes |
| xe96n92 | yes | No |
| li8if61 | cephalophes | duiker |
| li8if61 | potamocheres | Bushpig |
| li8if61 | pigs_epics | Porcupines |
| li8if61 | little_singes | Little monkeys |
| li8if61 | big_singes | Great apes (gorillas, chimpanzees) |
| li8if61 | bat_souris | Bats |
| li8if61 | pangolin | Pangolin |
| li8if61 | rat_palmiste | Palmetto rat |
| li8if61 | fox | Fox |
| li8if61 | herisson | Hedgehog |
| li8if61 | viper | Vipers |
| li8if61 | reptiles | Reptiles |
| li8if61 | toucan | Toucan |
| li8if61 | guinea fowl | Guinea fowl |
| li8if61 | chat_tigre | Tiger cat |
| li8if61 | cat_huant | Booing cat |
| li8if61 | chevrotin | Chevrotin |
| li8if61 | civet | Civet |
| li8if61 | crocodile | Crocodile |
| li8if61 | caiman | Caiman |
| li8if61 | pithon | Pithon |
| li8if61 | squirrel | Squirrel |

| | | |
|---|---|---|
| li8if61 | armadillo | Armadillo |
| li8if61 | elephant | Elephant |
| li8if61 | buffalo | Buffalo |
| li8if61 | other | Other |
| ho7ug89 | throw | Discard |
| ho7ug89 | destroy | Destroy |
| ho7ug89 | bury | Bury |
| ho7ug89 | consume | Keep for consumption |
| ho7ug89 | sell | Sell |
| ho7ug89 | other | Other |
| ho7ug89 | leave | leave |
| yi28g08 | yes | Yes |
| yi28g08 | no | No |
| un06u98 | family | Family members |
| un06u98 | hunters | Other hunters |
| un06u98 | villagers | Other villagers |
| un06u98 | chefs | Chefs |
| un06u98 | other | Other |
| bc6eh68 | yes | Yes |
| bc6eh68 | no | No |
| nh9de12 | consume | Take it to consume |
| nh9de12 | sell | Take it to sell it |
| nh9de12 | do_not_touch | Do not touch |
| nh9de12 | other | Other |
| nh9de12 | leave | leave |
| rp8me13 | yes | Yes |
| rp8me13 | no | No |
| vf5ez25 | family | Family members |
| vf5ez25 | hunters | Other hunters |
| vf5ez25 | villagers | Other villagers |
| vf5ez25 | chefs | Chefs |
| vf5ez25 | other | Other |
| rt6ep37 | yes | Yes |
| rt6ep37 | no | No |
| rd7yw62 | dog | Dog |
| rd7yw62 | cat | Cat |
| rd7yw62 | goat | Goat |
| rd7yw62 | sheep | Sheep |
| rd7yw62 | cattle | Cattle |
| rd7yw62 | pork | Pork |
| rd7yw62 | chicken | Chicken |
| rd7yw62 | other | Other |
| bq3pw07 | consume | Take it to consume |
| bq3pw07 | sell | Take it to sell it |
| bq3pw07 | do_not_touch | Don't touch it |
| bq3pw07 | other | Other |

| | | |
|---|---|---|
| bq3pw07 | leave | leave |
| pd4rh18 | yes | Yes |
| pd4rh18 | no | No |
| ie6te29 | family | Family members |
| ie6te29 | hunters | Other hunters |
| ie6te29 | villagers | Other villagers |
| ie6te29 | other | Other |
| rq9ny36 | yes | Yes |
| rq9ny36 | no | No |
| vz1cz24 | thinness | Lean |
| vz1cz24 | lethargie | Lethargy (slow movements) |
| vz1cz24 | cpt_anormal | Abnormal behavior |
| vz1cz24 | maggots | Presence of maggots |
| vz1cz24 | cayor | Presence of cayor worms |
| vz1cz24 | other | Other |
| sk5nq39 | consume | Capture for consumption |
| sk5nq39 | sell | Capture to sell |
| sk5nq39 | investigate_illness | Capture to investigate disease |
| sk5nq39 | do_not_touch | Don't touch it |
| sk5nq39 | other | Other |
| sk5nq39 | leave | leave |
| pf5kg71 | yes | Yes |
| pf5kg71 | no | No |
| rq0ut60 | family | Family members |
| rq0ut60 | hunters | Other hunters |
| rq0ut60 | villagers | Other villagers |
| rq0ut60 | chefs | Chefs |
| rq0ut60 | other | Other |
| gw3vu83 | yes | Yes |
| gw3vu83 | no | No |
| ae5dj10 | dog | Dog |
| ae5dj10 | cat | Cat |
| ae5dj10 | goat | Goat |
| ae5dj10 | sheep | Sheep |
| ae5dj10 | cattle | Cattle |
| ae5dj10 | pork | Pork |
| ae5dj10 | chicken | Chicken |
| ae5dj10 | other | Other |
| vn1ap87 | treat | Trying to cure it |
| vn1ap87 | insulate | Insulate it |
| vn1ap87 | slaughter_and_consume | Slaughter for consumption |
| vn1ap87 | sell | Sell it |
| vn1ap87 | other | Other |
| vn1ap87 | leave | leave |
| ec5jk61 | yes | Yes |
| ec5jk61 | no | No |

| | | |
|---|---|---|
| el45z22 | family | Family members |
| el45z22 | hunters | Other hunters |
| el45z22 | villagers | Other villagers |
| el45z22 | other | Other |
| iq9zk31 | yes | Yes |
| iq9zk31 | no | No |
| iq9zk31 | did_not_know | Don't know |
| zq3la00 | yes | Yes |
| zq3la00 | no | No |
| zq3la00 | did_not_know | Don't know |
| fn4je65 | yes | Yes |
| fn4je65 | no | No |
| am9vk61 | yes | Yes |
| am9vk61 | no | No |
| am9vk61 | did_not_know | Don't know |
| sd0pf73 | contact_physique | Physical contact with a live animal |
| sd0pf73 | bite | Animal bite |
| sd0pf73 | cleaning | Butchering an animal |
| sd0pf73 | fresh_meat_handling | Fresh meat handling |
| sd0pf73 | sauerkraut_handling | Handling smoked meat |
| sd0pf73 | conso_meat_cooked | Cooked meat consumption |
| sd0pf73 | conso_product_cru | Consumption of raw animal products |
| or1tw70 | yes | Yes |
| or1tw70 | no | No |
| or1tw70 | did_not_know | Don't know |
| ac5bt15 | measure_1 | Measure 1 |
| ac5bt15 | measure_2 | Measure 2 |
| mu3xg98 | yes | Yes |
| mu3xg98 | no | No |
