# Supplementary material for: Co-designing zoonotic diseases prevention practices when people depend on wild meat
Source: One Health. 2025 May 13;20:101074. doi: 10.1016/j.onehlt.2025.101074 (PMC12152596; doi:10.1016/j.onehlt.2025.101074)
Supplement: Supplementary file 2 — Appendix B: Additional results of the questionnaire survey on zoonotic risk knowledge and practices. [file mmc2.pdf]

Supplementary information S2. Additional results of the Knowledge-Attitude-Practice questionnaire survey

1. Storage of animal products

191 of the 287 respondents involved in the storage of wild animal products at the village reported preserving a fraction of the wild animal products fresh and 191 of the 287 respondents reported conserving it smoked, a fraction of the respondents using both methods alternatively. Storing of fresh products in freezers was reported by 188 respondents. The storage of wild animal products at the campsite for a short period of time (a few days) was described by 78 hunters who used a campsite in their hunting trip. 23 and 69 of them reported storing the wild animal products at the camp fresh (in the open air or in the river) and smoked respectively, some respondents reporting both methods.

2. Disposal of unconsumed animal parts

10% (n=24) of participants involved in butchering reported the disposal of unconsumed animal parts, and 62% (n=15) of them disposed of it in the environment or in water ponds instead of garbage containers. 63% (n=182) of participants involved in the cooking reported the disposal of unconsumed animal parts, and 33% (n=60) of them disposed of it in the environment or in water ponds instead of garbage containers.

3. Perception of zoonotic diseases and preventive measures

The zoonotic diseases most frequently cited by respondents included Ebola (n=76), coronaviruses (n= 14), rabies (n=14), and avian influenza (n=10). The wild animal categories most frequently cited as sources of zoonotic disease infection were apes (n=65), monkeys (n=62), and duikers (n=40). Most of the participants who knew at least one protective measure cited avoidance behaviour, like the avoidance of contacts with live animals of animals found dead (n=97) while a minority cited proactive behaviours like the proper smoking or cooking of the products (n=24), vaccination (n=5) or handwashing (n=4).
