# Supplementary material for: Co-designing zoonotic diseases prevention practices when people depend on wild meat
Source: One Health. 2025 May 13;20:101074. doi: 10.1016/j.onehlt.2025.101074 (PMC12152596; doi:10.1016/j.onehlt.2025.101074)
Supplement: Supplementary file 3 — Appendix C: Poster displaying the main recommendations to reduce the risk of disease transmission from animals to humans. [file mmc3.pdf]

cirad

Organisation des Nations Unies pour l'alimentation et l'agriculture

World Agroforestry

WCS

PROGRAMME DE GESTION DURABLE DE LA FAUNE SAUVAGE
SWM PROGRAMME

BONNES PRATIQUES POUR LIMITER LE RISQUE DE TRANSMISSIONS DE MALADIES DES ANIMAUX AUX ETRES HUMAINS

CHASSE

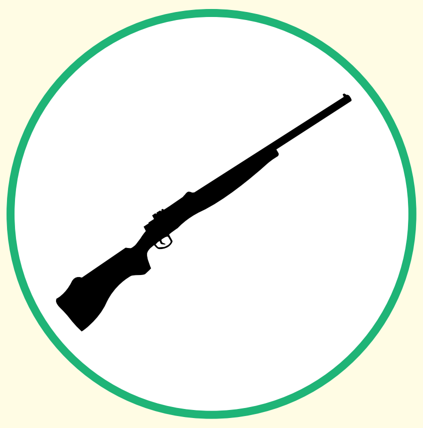

Tuer les animaux au fusil est le meilleur moyen de les abattre et de limiter les blessures

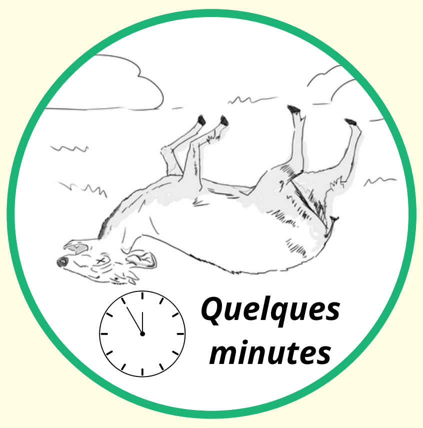

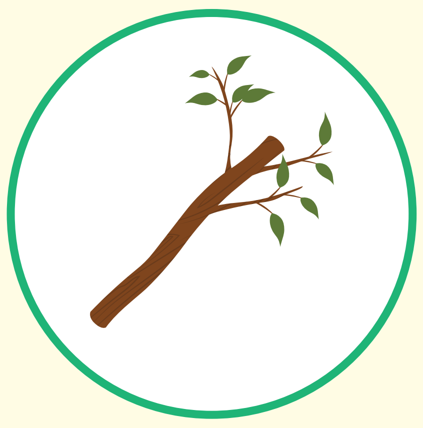

Avant de s'approcher d'une bête étendue sur le sol, attendre quelques instants et vérifier que celle-ci est bien morte pour limiter les blessures

-

La vérification peut être faite à l'aide d'un long bois

CAPTURE D'ANIMAUX VIVANTS

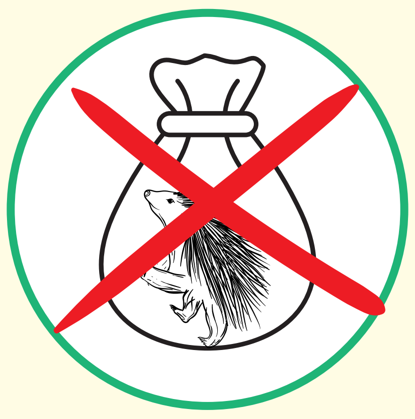

Limiter la capture et le transport des animaux vivants

TRANSPORT

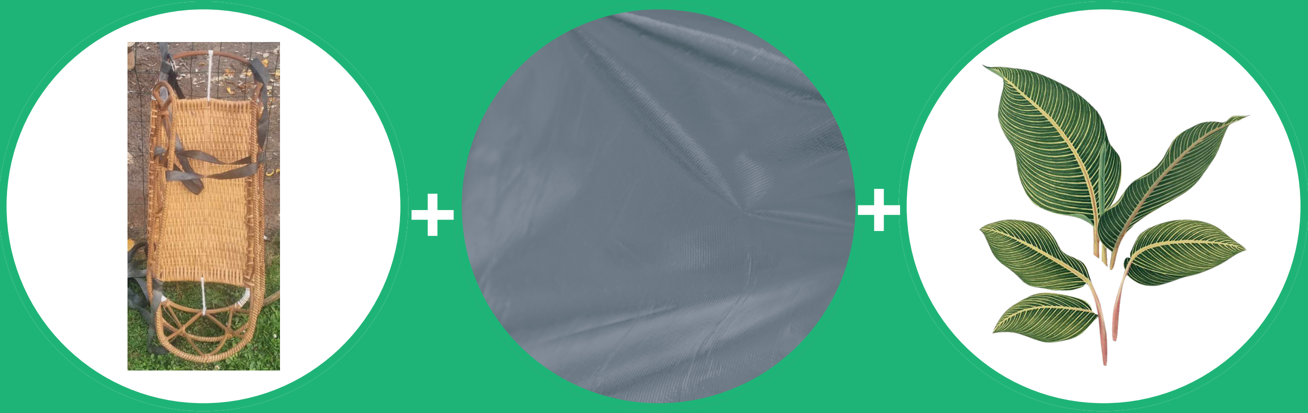

Combiner la hotte, la polyandre et les feuilles

-

La fraicheur du gibier sera assurée par les feuilles et la polyandre protégera le porteur des fluides corporels (du sang, du pipi, ...)

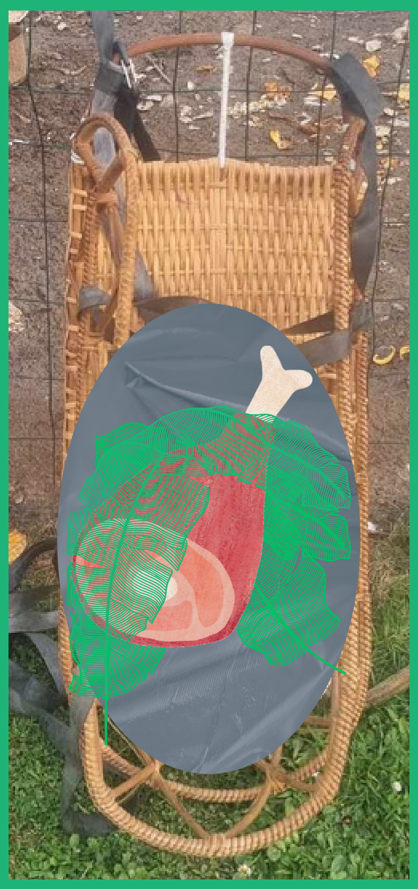

DEPECAGE ET DECOUPE DE GIBIER

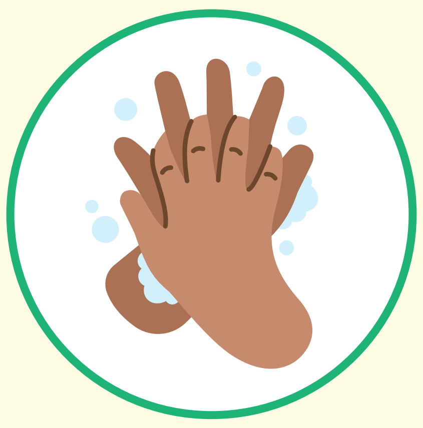

Après avoir touché la viande fraîche (ou fumée), se laver les mains systématiquement avec de l'eau et du savon

PERCEPTION DU RISQUE & RENCONTRES DE CAS SUSPECTS

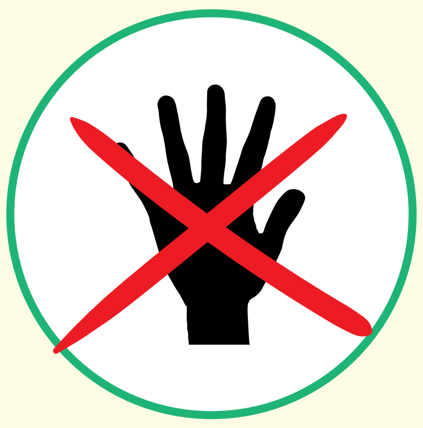

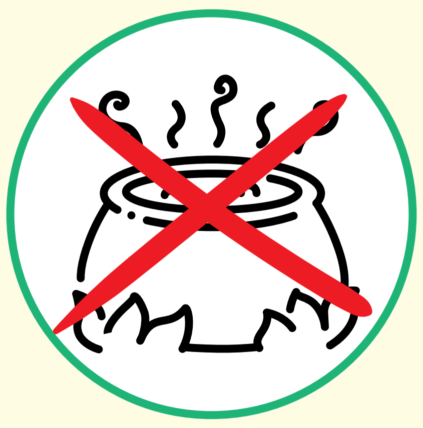

Ne pas toucher, ne pas consommer

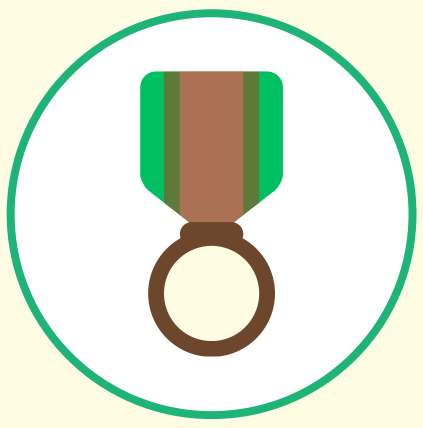

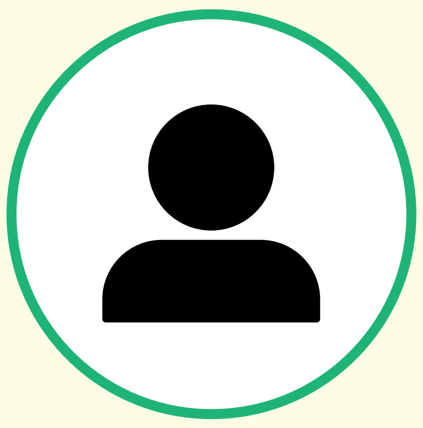

Saisir un responsable (Chef ou relais communautaire)

Dessin gazelle : B. Konradowski

Avec le soutien de

Cofinancé par l'Union européenne

www.swm-programme.info

Partenaires locaux

CIRMF GABON

IRET

EAUX ET FORETS
