# Supplementary material for: Co-designing zoonotic diseases prevention practices when people depend on wild meat
Source: One Health. 2025 May 13;20:101074. doi: 10.1016/j.onehlt.2025.101074 (PMC12152596; doi:10.1016/j.onehlt.2025.101074)
Supplement: Supplementary file 4 — Appendix D: Informed consent forms. [file mmc4.pdf]

Assessment of current practices of hunting and handling of wild animals, and perception and knowledge of zoonotic risks in the communities

Informed consent form

Assessment of the practices and knowledge of wild meat supply chain operators regarding potential zoonotic risks

2023

Project team members:

Natacha Efoua Tomo

Aude Pouliquen

Gilles Boupana

Alexis Delabouglise

Marie-Marie Olive

Gael Maganga

Purpose of the study:

We invite you to take part in an interview-based research project. Informed consent is required from participants.

Before you agree to take part in this interview, please take the time to read the following information so that you know whether you want to participate or not.

This interview is part of the SWM (Sustainable Wildlife Management) program, funded by the European Union, with the aim of better understanding current wildlife management and implementing sustainable management. The information is being collected by CIRAD (Centre de Coopération Internationale de Recherche Agronomique pour le Développement), a research organization based in Montpellier, France, and CIRMF (Centre Interdisciplinaire de Recherche Médicale de Franceville), a Gabonese research organization based in Franceville in the Haut Ogooué province.

The aim of this study is to assess the state of practices and knowledge of wild meat supply chain actors regarding the risks associated with hunting and handling game. This study is a prerequisite to the implementation of a surveillance system for the early detection of zoonotic diseases in five pilot hunting communities participating in the SWM program.

You are free to decide whether or not to take part in this survey, and whether or not to answer one or more questions.

Your participation is voluntary and free of charge, and will have no negative consequences as your answers are confidential.

Data protection:

The data are collected by CIRMF and CIRAD, who are jointly responsible for processing.

The information collected is for the purpose of conducting a survey to assess the state of practices and knowledge of those involved in the wild meat supply chain regarding the health risks associated with hunting and handling game.

This processing is based on your explicit consent, expressed on the "participant form" that you will be asked to sign before the start of the interviews.

Analyzed data will be kept until the end of the study, when they will be anonymized. For publications, the names, locations and contacts of participants will not be mentioned. Members of the project team reserve the right to publish data once they have been fully anonymized.

This information is intended for use by the CIRAD and CIRMF research teams, on condition of confidentiality and solely within the framework of the aforementioned project.

In accordance with the applicable regulations, in particular the RGPD (Regulation (EU) 2016/679 of the European Parliament and of the Council of 27 April 2016 on the protection of individuals with regard to the processing of personal data and on the free movement of such data), you have the right to access, rectify, delete and port your personal data (where applicable), as well as the right to limit and object to their processing for legitimate reasons. You can exercise these rights by contacting CIRAD's Data Protection Officer at XXXXX or CIRMF at XXXXX. You also have the right to lodge a complaint with the competent authority (in France, the CNIL) at any time.

**Data**	**Shelf life**	**Access conditions**	**Explanation**
Informed consent form	8 years (2031)	CIRMF researchers; CIRAD and SWM program partners; publishers	Archived in case of audit
Anonymized survey data	Unlimited (because anonymous)	CIRMF researchers; CIRAD and SWM program partners;<br><br>The database may be made public once it has been fully anonymized.	Data anonymization
Results	Unlimited (because aggregated and anonymous)	No restrictions	No personal data

If you have any questions, please contact Gaël MAGANGA (email address and number XXXXX) and Alexis DELABOUGLISE (email address and number XXXXX).

Participant sheet

Interrupting the interview:

The maximum duration of the interview is two hours. Participation in the interview is voluntary, and once you have given your consent, you can always ask for a break during the interview, or interrupt it at any time.

Survey participation:

Your participation consists of: taking part in the interview, answering the interviewer's questions.

Project	Assessment of wild meat industry players' practices and knowledge of potential zoonotic risks
Lead researcher	Alexis Delabouglise
Person interviewed	
Interviewer	
Date and place of interview	

	YES	NO
1. Did you receive and understand the information you were given?		
2. Were you able to ask questions?		
3. Do you understand that you can decide to stop taking part in the survey at any time?		
4. Do you agree to take part in the study?		
5. Do you understand your rights regarding your personal data?		
6. Do you agree to the use of the data collected in this survey for the purposes, retention period and recipient indicated in the information letter?		
7. Do you agree that the research team may contact you again for a subsequent interview?		

<table>
<tr><td colspan="2">Participant's signature</td><td colspan="2"></td></tr>
<tr><td rowspan="2"></td><td rowspan="2">If signing is not possible</td><td>Witnessed verbal consent</td><td></td></tr>
<tr><td>Name of witness</td><td></td></tr>
<tr><td colspan="2">Investigator's signature</td><td colspan="2"></td></tr>
</table>

Co-design of risk-reduction practices

Informed consent form

Research into alternative solutions to practices that present risks of transmitting pathogens from animals to humans, from tracking animals to eating game.

2023

Project team members:

Natacha Efoua Tomo

Aude Pouliquen

Gilles Boupana

Alexis Delabouglise

Marie-Marie Olive

Gael Maganga

Purpose of the study:

We invite you to take part in an interview-based research project. Informed consent is required from participants.

Before you agree to take part in this interview, please take the time to read the following information so that you know whether you want to participate or not.

This interview is taking place within the framework of the SWM (Sustainable Wildlife Management) program, funded by the European Union, with the aim of better understanding current wildlife management and implementing sustainable management. The information is being collected by CIRAD (Centre de Coopération Internationale de Recherche Agronomique pour le Développement), a research organization based in Montpellier, France, and CIRMF (Centre Interdisciplinaire de Recherche Médicale de Franceville), a Gabonese research organization based in Franceville in the Haut Ogooué province.

The aim here is to exchange ideas with members of the community to propose changes in practices that will lead to better sanitary management of hunting, transport, butchering, conservation and consumption of hunting products.

You are free to participate or not in this exchange.

Your participation is voluntary and free of charge, and will have no negative consequences.

Data protection:

The data are collected by CIRMF and CIRAD, who are jointly responsible for processing.

The information collected will be used to propose alternative solutions to practices considered risky, i.e. those that encourage the transmission of pathogens from animals to humans. Observation notes and recordings will be made of interviews to facilitate the collection of information.

This processing is based on your explicit consent, expressed on the "participant form" that you will be asked to sign before the start of the interviews.

Analyzed data will be kept until the end of the study, when they will be anonymized. For publications, the names, locations and contacts of participants will not be mentioned. Members of the project team reserve the right to publish data once they have been fully anonymized.

This information is intended for use by the CIRAD and CIRMF research teams, on condition of confidentiality and solely within the framework of the aforementioned project.

In accordance with the applicable regulations, in particular the RGPD (Regulation (EU) 2016/679 of the European Parliament and of the Council of 27 April 2016 on the protection of individuals with regard to the processing of personal data and on the free movement of such data), you have the right to access, rectify, delete and port your personal data (where applicable), as well as the right to limit and object to their processing for legitimate reasons. You can exercise these rights by contacting CIRAD's Data Protection Officer at XXXXX or CIRMF at XXXXX. You also have the right to lodge a complaint with the competent authority (in France, the CNIL) at any time.

Data	Shelf life	Access conditions	Explanation
Informed consent form	8 years (2031)	CIRMF researchers; CIRAD and SWM program partners; publishers	Archived in case of audit
Recordings	3 months	Investigators only	Transcription time does not contain personal information
Anonymized survey data	Unlimited (because anonymous)	CIRMF researchers; CIRAD and SWM program partners;<br><br>The database may be made public once it has been fully anonymized.	Data anonymization
Results	Unlimited (because aggregated and anonymous)	No restrictions	No personal data

If you have any questions, please contact Gaël MAGANGA (email address and number XXXXX) and Alexis DELABOUGLISE (email address and number XXXXX).

Participant sheet

Interrupting the interview:

The maximum duration of the interview is two hours. Participation in the interview is voluntary, and once you have given your consent, you can always ask for a break during the interview, or interrupt it at any time.

Survey participation:

Your participation consists of: taking part in the interview, answering the interviewer's questions.

Project	Research into alternative solutions to practices that present risks of transmitting pathogens from animals to humans, from tracking animals to eating game.
Lead researcher	Alexis Delabouglise
Person interviewed	1.<br>2.<br>3.<br>4.<br>5.<br>6.<br>7.<br>8.<br>9.<br>10.<br>11.<br>12.<br>13.<br>14.<br>15.
Interviewer(s)	Natacha Efoua Tomo and Aude Pouliquen
Date and place of interview	

	YES	NO
8. Did you receive and understand the information you were given?		
9. Were you able to ask questions?		
10. Do you understand that you can decide to stop taking part in the survey at any time?		
11. Do you agree to take part in the study?		
12. Do you understand your rights regarding your personal data?		

13. Do you agree to the use of the data collected in this survey for the purposes, retention period and recipient indicated in the information letter?		
14. Do you agree that the research team may contact you again for a subsequent interview?		

<table>
<tr><td colspan="2">Participant's signature</td><td colspan="3"></td></tr>
<tr><td rowspan="2"></td><td rowspan="2">If signing is not possible</td><td colspan="2">Witnessed verbal consent</td><td></td></tr>
<tr><td>Name of witness</td><td colspan="2"></td></tr>
<tr><td colspan="2">Investigator's signature</td><td colspan="3"></td></tr>
</table>
